# Leveraging artificial intelligence to improve people’s planning strategies

**DOI:** 10.1073/pnas.2117432119

**Published:** 2022-03-16

**Authors:** Frederick Callaway, Yash Raj Jain, Bas van Opheusden, Priyam Das, Gabriela Iwama, Sayan Gul, Paul M. Krueger, Frederic Becker, Thomas L. Griffiths, Falk Lieder

**Affiliations:** ^a^Department of Psychology, Princeton University, Princeton, NJ 08540;; ^b^Rationality Enhancement Group, Max Planck Institute for Intelligent Systems, 72076 Tübingen, Germany;; ^c^Department of Cognitive Sciences, University of California, Irvine, CA 92697-5100;; ^d^Department of Psychology, University of California, Berkeley, CA 94720-1650;; ^e^Department of Computer Science, Princeton University, Princeton, NJ 08540

**Keywords:** cognitive training, bounded rationality, heuristics, rationality enhancement

## Abstract

Human decision making is plagued by systematic errors that can have devastating consequences. Previous research has found that such errors can be partly prevented by teaching people decision strategies that would allow them to make better choices in specific situations. Three bottlenecks of this approach are our limited knowledge of effective decision strategies, the limited transfer of learning beyond the trained task, and the challenge of efficiently teaching good decision strategies to a large number of people. We introduce a general approach to solving these problems that leverages artificial intelligence to discover and teach optimal decision strategies. As a proof of concept, we developed an intelligent tutor that teaches people the automatically discovered optimal heuristic for environments where immediate rewards do not predict long-term outcomes. We found that practice with our intelligent tutor was more effective than conventional approaches to improving human decision making. The benefits of training with our cognitive tutor transferred to a more challenging task and were retained over time. Our general approach to improving human decision making by developing intelligent tutors also proved successful for another environment with a very different reward structure. These findings suggest that leveraging artificial intelligence to discover and teach optimal cognitive strategies is a promising approach to improving human judgment and decision making.

## Significance

Many bad decisions and their devastating consequences could be avoided if people used optimal decision strategies. Here, we introduce a principled computational approach to improving human decision making. The basic idea is to give people feedback on how they reach their decisions. We develop a method that leverages artificial intelligence to generate this feedback in such a way that people quickly discover the best possible decision strategies. Our empirical findings suggest that a principled computational approach leads to improvements in decision-making competence that transfer to more difficult decisions in more complex environments. In the long run, this line of work might lead to apps that teach people clever strategies for decision making, reasoning, goal setting, planning, and goal achievement.

Planning skills are generally beneficial to the wellbeing of individuals (1) and the success of organizations (2). But many people frequently fail to plan (3, 4) and consequently make bad decisions (5). Furthermore, even when people do plan, they often use short-sighted planning strategies that prevent them from achieving the best long-term outcomes (4).

Previous research on improving human decision making has found that incentivizing or motivating people to make better decisions is not enough, because people sometimes lack effective decision strategies (6, 7). People can learn more effective strategies through practice (8–11), but experience is a good teacher only to the extent that it provides reliable, valid, and prompt feedback (12–15). This is not the case in many important real-life settings including financial investment, college admissions, and the diagnosis of mental disorders (16).

To provide effective strategies, many interventions have instructed people in the normative principles of logic, probability, and expected utility theory (17)—principles that human decisions and judgments have been found to violate (7, 18–20). This approach has had limited success, however, because trying to calculate the expected utilities of all possible courses of actions is actually not a good decision strategy in complex real-life situations where it is often prohibitively difficult and time consuming (21, 22). Subsequent work has therefore sought to identify and teach simple heuristics that exploit common properties of certain types of decision problems to quickly reach a good decision most of the time (23, 24).

Despite some initial success, teaching people clever heuristics suffers from two bottlenecks that we attempt to address in this work. The first one is the identification of simple heuristics that reliably lead to good decisions, a process that can itself be error prone and time consuming. To overcome this limitation, we employ artificial intelligence to automatically discover optimal heuristics (25). Given a formal characterization of a decision maker’s environment and cognitive limitations, this method derives an optimal heuristic for decision making.

The second bottleneck is efficiently teaching clever heuristics to a large number of people. To overcome this bottleneck, we introduce a general method that can be applied to create desktop, web, and mobile applications for practicing decision making with feedback. The basic idea is to have people practice on relevant real-world tasks or simulations of those tasks while giving them feedback on how they solve those tasks. Previous research has shown that, to be effective at furthering the acquisition of expertise, feedback has to be valid, reliable, and prompt (13, 16). We introduce a general method for generating high-quality feedback for helping people learn how to make better decisions. The central idea is to give people feedback on how they decide what to do (metacognitive feedback) rather than on what they decide to do (action feedback) (26). Giving metacognitive feedback is possible when we can infer the decision operations that people perform from overt behavior such as sequentially looking at different pieces of information that inform their decision (27). As people make more and more decisions at their computers and smartphones, there is an increasingly larger range of decisions for which metacognitive feedback can be given automatically. This suggests that the idea of giving people metacognitive feedback could be applied to develop a scalable approach to improving human decision making.

As a proof of concept, we develop an internet-based cognitive tutor that helps people learn and practice optimal heuristics for solving sequential decision problems. Those heuristics are automatically derived by applying our recently developed artificial intelligence method for strategy discovery to an environment in which long-term consequences are more important than immediate rewards (25). Encouragingly, we found that practice with our cognitive tutor was more effective at promoting far-sighted decision-making than conventional approaches to improving human decision-making. Concretely, practicing with our intelligent tutor improved learning relative to practice without feedback and practice with feedback on the chosen actions rather than on the decision process itself. Critically, we also found that the benefits of training with our cognitive tutor transferred to more complex and superficially different tasks, and these benefits were retained over time. Finally, we illustrate the generality of our approach by applying it to an environment with a different structure, again finding a benefit of practicing with the tutor above practice alone. Together, these findings suggest that leveraging artificial intelligence to discover and teach optimal cognitive strategies is a promising approach to improving human judgment and decision making.

## A Principled Computational Approach to Improving Human Decision Making

Our approach to cognitive training teaches people optimal decision strategies by giving them metacognitive feedback throughout the decision process leading to a single choice. Since recent findings suggest that people acquire, refine, and learn to select between their cognitive strategies at least partly through reinforcement learning (10, 15, 28, 29), we address the question of what constitutes an optimal metacognitive framework from a reinforcement learning perspective. That is, we develop a general method for computing the metacognitive feedback that results in the fastest possible learning according to the recently developed theory of metacognitive reinforcement learning (10, 15, 28, 29). Our approach is based on four building blocks that are introduced in turn: 1) making people’s planning strategies observable, 2) simulating challenging decision problems, 3) discovering optimal strategies for solving those problems, and 4) giving people feedback on their planning operations and on what the optimal planning strategy might have done differently.

### 1) Making People’s Planning Strategies Observable.

**Fig. 1.**
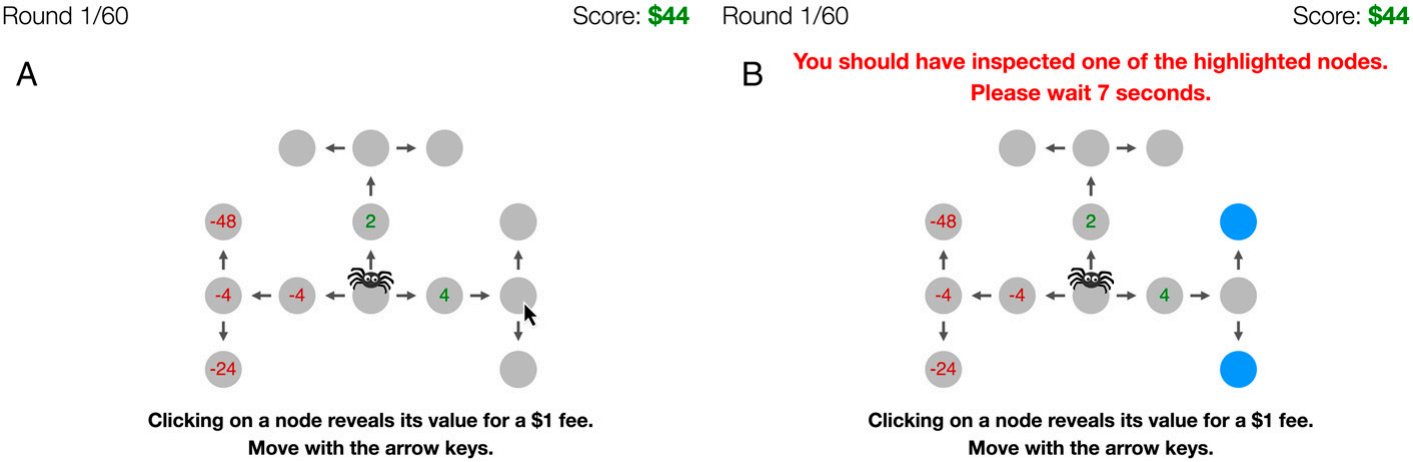
The Mouselab-MDP paradigm. (*A*) Participants click to reveal the rewards at future states to construct a plan. (*B*) Metacognitive feedback penalizes suboptimal decision-making operations (clicks) with a delay and provides instruction on what operation(s) should have been taken instead.

Giving people feedback on their decision strategies is challenging when we cannot observe how they actually made those decisions. Thus, we employ a recently developed process-tracing paradigm that makes the decision-making process observable (30): The Mouselab-MDP paradigm extends the process-tracing methodology of the Mouselab paradigm (27) from risky choice to sequential decision problems that require planning, that is, Markov decision processes (MDPs) (31). The participants’ task is to navigate a spider through a web (Fig. 1*A*) by selecting a sequence of moves that leads from its initial location at the center of the web to one of its corners. Each location contains a reward, and the participant’s goal is to earn as much reward as possible. Critically, all of the rewards are initially concealed. To uncover a reward, the participant has to click on its location and pay a small fee. In this way, an internal planning process is externalized as a sequence of information-gathering clicks. This allows us to separate what people choose to do (i.e., where they move the spider) from how they decide to do it (i.e., by clicking to reveal a subset of the available information in a specific order). In other words, we treat the clicks as a proxy for the cognitive operations that people perform to reach a decision.

### 2) A Simple Task for Practicing Far-Sighted Decision Making.

The key property of situations that necessitate planning is the misalignment between immediate reward and long-term value. As an illustration of this problem, consider the choice between beginning work on a manuscript versus watching a YouTube video. Staring at a blank page might make one feel anxious in the short run, but one will feel very satisfied when one submits the paper for publication many months later. By contrast, the YouTube video will give one immediate joy but one might come to regret the wasted time later. To make good decisions in situations like this, people have to look beyond the salient immediate rewards, set a goal for the future, plan how to achieve it, and execute the plan. What makes this far-sighted approach worthwhile is that the range of outcomes that can be obtained by concerted effort over an extended period of time is much larger than the range of rewards that can be attained immediately.

To capture this aspect of many real-world situations within the Mouselab-MDP paradigm, we constructed a three-step sequential decision-making task where the range of rewards increases from the first step to the second step and is largest in the third step. In each trial, rewards are independently drawn from discrete uniform distributions; the possible values are {−4,−2,+2,+4} in the first step, {−8,−4,+4,+8} in the second step, and {−48,−24,+24,+48} in the third step. To simulate the computational cost of deliberation, which is substantially diminished in this externalized planning problem, we impose a $1 cost for each click.

### 3) Discovering Optimal Cognitive Strategies.

Teaching clever heuristics is a promising approach to improving decision making (23, 24). But which heuristics should be taught and how can we discover such heuristics? The theory of resource rationality provides a mathematically precise definition of optimal heuristics (32). In essence, the optimal heuristic for a decision maker to use in a given environment is the one that achieves the best possible tradeoff between the expected utility of the resulting decision and the expected cost of the decision-making process.

To derive the optimal heuristic for the Mouselab-MDP environment described above, we apply the recently developed formalism of metalevel MDPs (33, 34), which models decision making itself as a sequential decision problem. The basic idea is that the decision-making process can be broken down into a series of computations that update the decision maker’s beliefs about which course of action will lead to the best outcome. Each cognitive strategy, or heuristic, corresponds to a rule for selecting computations based on the outcome of previous computations.

Formally, a metalevel MDP, $M_{\text{meta}}=(B,C,T_{\text{meta}},r_{\text{meta}})$, has four components: the set of possible beliefs the decision maker can have, B; the set of computations they can perform, C; the transition model that specifies how computations update beliefs, $T_{\text{meta}}$; and the metalevel reward function that specifies the cost of computation and the expected utility of making a final decision in a given belief state, $r_{\text{meta}}$. A cognitive strategy can be formalized as a metalevel policy, $\pi_{\text{meta}}:B\mapsto C$, that specifies which computation should be performed in each belief state.

Having formalized decision making as a Markov decision process, we can use standard MDP-solving techniques (35) to identify optimal decision strategies. One important such tool is the state-action value function (often notated *Q*). In a metalevel MDP, this function gives the long-term expected value of performing a computation *c* in belief state *b*. It is defined as $Q_{\text{meta}}(b,c)=E\left[r_{\text{meta}}(b,c,b\prime )+{\text{max}}_{c\prime }Q_{\text{meta}}(b\prime ,c\prime )\right]$, where b′ is the updated belief that results from executing computation *c* given the belief *b*. The optimal decision strategy is the policy that always executes the most valuable decision operation; i.e., $\pi_{\text{meta}}^{⋆}(b)={\text{argmax}}_{c}Q_{\text{meta}}(b,c)$. Following previous work, we compute $Q_{\text{meta}}$ for our environment by backward induction (25). This provides us with both the optimal metalevel policy and a way to quantify exactly how bad is a specific deviation from the optimal policy.

This method revealed that the resource-rational heuristic for the environment described above is to first set a goal by evaluating potential final destinations. As soon as one uncovers the highest possible reward (+ 48), the optimal heuristic immediately selects the path leading to it, not even uncovering the values that will be received along the way. If all potential final destinations have been inspected and one was revealed to be better than all the others, then the optimal heuristic immediately decides to go there; otherwise, it works backward until one path is revealed to be better than the alternatives.

### 4) An Optimal Feedback Method for Teaching Planning Strategies.

If people acquire planning skills through metacognitive reinforcement learning (10, 15, 29), then it should be possible to apply methods that have been developed to accelerate model-free reinforcement learning in robots—such as reward shaping (36)—to accelerate metacognitive learning in people. Here, we apply reward shaping to generate optimal feedback signals for accelerating metacognitive reinforcement learning as follows:

1) Model the cognitive function to be improved (i.e., planning) and the available cognitive operations (i.e., determining the outcome of taking a certain action in a certain state) and their costs as a metalevel MDP, $M_{\text{meta}}$.
2) Compute the values of the planning operations people might perform in different states [i.e., $Q_{\text{meta}}(b,c)$] by solving the metalevel MDP, $M_{\text{meta}}$.
3) Let people practice planning and infer their decision process from their clicks.
4) Evaluate each inferred decision operation, *c*, by
  [1] $$\text{loss}(b,c)=\underset{{}_{c\prime }}{\max}Q_{\text{meta}}(b,c\prime )-Q_{\text{meta}}(b,c).$$
5) Translate the loss into reinforcement and inform the tutee what operation(s) should have been selected instead, i.e., $\arg\;\max_{c}Q_{\text{meta}}(b,c)$.

We completed steps 1 and 2 in previous work (25). Step 3 is accomplished by using the Mouselab-MDP paradigm to measure people’s planning operations. Finally, the feedback signal computed in step 4 is translated into a delay penalty and the states that would have been optimal to click are highlighted (Fig. 1*B*). Feedback is given after each click and also when the participant first moves the spider. Moving the spider when one should have clicked or vice versa incurs a penalty as well. See *SI Appendix*, Fig. S8 and *Materials and Methods* for details.

## Results

By combining the four building blocks described above, we created an intelligent cognitive tutor that employs metacognitive feedback to teach far-sighted planning. It does so by teaching them the optimal planning strategy for an environment in which distal outcomes are more important than proximal ones. We evaluated the effectiveness of this cognitive tutor in a series of six experiments, each comprising a training block in which the experimental group worked with the cognitive tutor and a test block in which all participants solved the same planning problems without feedback. We found that practice with our intelligent tutor was more effective than conventional approaches (experiment 1) and led to transferable improvements in decision making (experiment 2) that are retained over time (experiment 3).

To illustrate the generality of our approach, we created a second intelligent tutor—this time for an unstructured environment in which proximal and distal outcomes are equally important. As before, we found that practice with the new tutor was more effective than practice alone (experiment 4). In experiment 5, we found that the benefits of metacognitive feedback transfer to problems that are superficially dissimilar from the training task. Finally, experiment 6 investigated the relative contributions of the affective and informative components of the tutor’s metacognitive feedback. We found that both components are likely to contribute but that the affective component is especially critical.

### Experiment 1: Metacognitive Feedback Is Most Effective.

Experiment 1 evaluated the efficacy of our intelligent tutor’s metacognitive feedback against the conventional approaches of giving people feedback on their actions (e.g., “You should have gone left”) or having them practice without feedback. The training block and the test block both employed the three-step planning task shown in Fig. 1*A*.

To quantify participants’ task performance, we define “relative test score” as the average score each participant achieved in the test block, normalized by chance and optimal performance such that 0 points is chance and 100 points is optimal (Eq. **2**). People’s performance was strongly bimodal (*SI Appendix*, Fig. S1*A*), violating the distributional assumptions of parametric hypothesis tests; we therefore employed the nonparametric Kruskal–Wallis ANOVA and permutation tests (37) to analyze our participants’ scores. All analyses are conducted at the participant level.

**Fig. 2.**
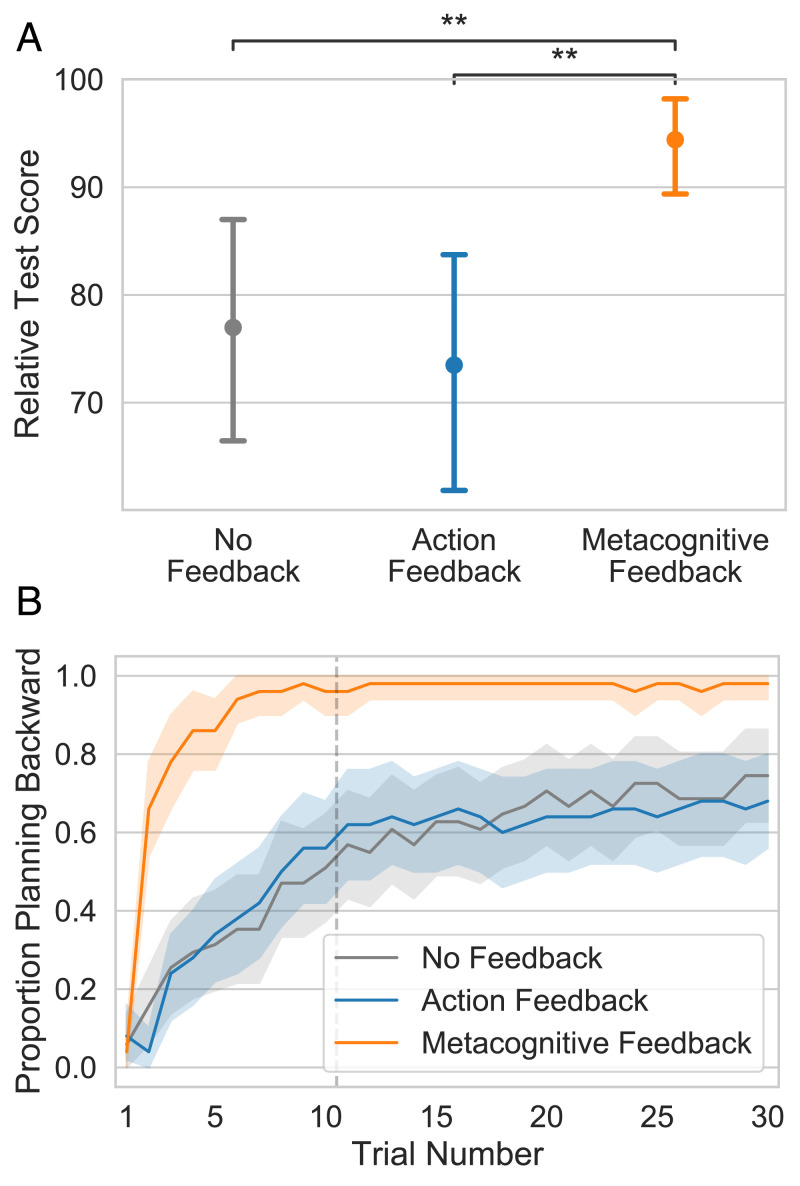
Metacognitive feedback accelerates learning and improves performance. (*A*) Average score in the test block for each condition. (*B*) Proportion of participants who started by inspecting a potential final outcome split by condition. Here and in all future plots, the error bars and shaded areas convey 95% confidence intervals produced by 1,000 bootstrap samples. Scores are bootstrapped over participant means. Asterisks indicate significance of the permutation test reported in the main text as follows: **P < 0.01.

Fig. 2*A* shows the average relative test score achieved by participants in each group. A Kruskal–Wallis ANOVA confirmed that the type of feedback provided in the training trials had a significant effect on participant performance (H=9.71,P=0.008). Participants who received no feedback achieved an average relative test score of 77.0 points (95% CI [66.2, 86.8]). This means they achieved 77% of the possible increase in score that one could gain by planning rather than choosing a path randomly. Critically, participants receiving metacognitive feedback performed significantly better, coming close to optimal performance (94.4 points; 95% CI [89.1, 98.5], permutation test d=0.59,Z=2.84,P=0.005). By contrast, giving participants conventional feedback on their actions appeared to be ineffective. That is, participants receiving action feedback did not perform better than participants in the no-feedback condition (73.5 points; 95% CI [61.8, 83.9], d=−0.09,Z=−0.45,P=0.653) and performed significantly worse than participants who received metacognitive feedback (d=0.68,Z=3.22,P=0.001).

To identify the mechanism by which metacognitive feedback improved performance, we conducted a causal mediation analysis (38). We found that the effect of metacognitive feedback on performance was fully mediated by an increase in people’s propensity to start by inspecting a potential final outcome (average causal mediation effects 21.9 points, 95% CI [12.0, 33.3], *P* < 0.001; average direct effects –4.2 points, 95% CI [–13.3, 4.9], *P* = 0.346). Metacognitive feedback—but not action feedback—significantly increased participants’ propensity to plan backward [t(148)=4.27, *P* < 0.001 for metacognitive feedback; t(148)=−0.21, *P* = 0.831 for action feedback]. Specifically, participants who received metacognitive feedback planned backward on 97.7% of test trials compared to 66.1% in the no-feedback group and 64.5% in the action-feedback group. Backward planning, in turn, increased participants’ average performance by 67.0 points [t(149)=15.02, *P* < 0.001]. As shown in Fig. 2*B*, people gradually learned to plan backward in all three conditions but the metacognitive feedback significantly boosted this learning process. Overall, our findings suggest that metacognitive feedback was effective because it taught participants a simple, clever heuristic that allowed them to make better decisions without having to think harder.

### Experiment 2: Transfer.

**Fig. 3.**
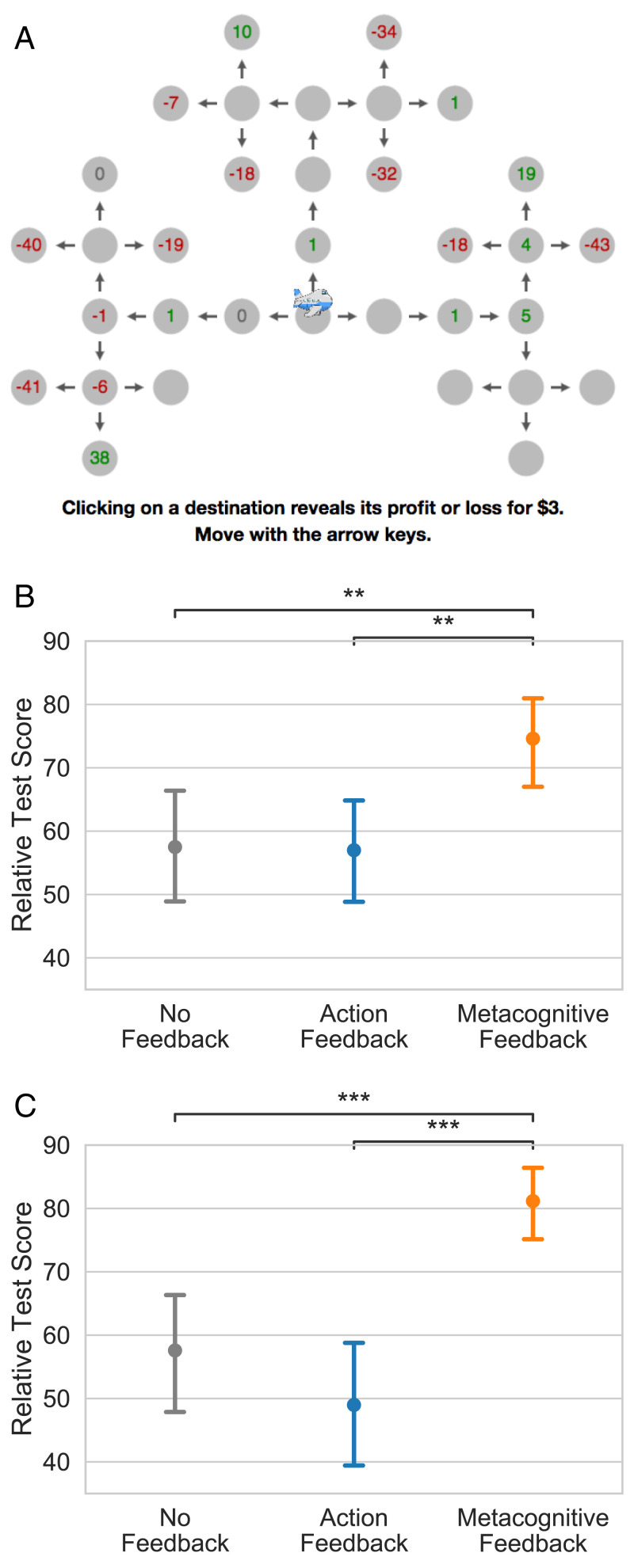
The benefits of metacognitive feedback transfer to more difficult problems and are retained for at least 24 h. (*A*) The near-transfer task is a five-step sequential decision problem where the rewards are normally distributed with a variance that increases exponentially from the first step to the last step. (*B*) Average performance on the transfer task given immediately after training. (*C*) The same, but with a 24-h delay between training and test. **P < 0.01, ***P < 0.001.

Experiment 2 examined whether the benefits of the strategy training evaluated in experiment 1 transfer to a more complex task. The training block was the same as in experiment 1 but the test block used the more complex flight-planning task illustrated in Fig. 3*A*. In this transfer task, participants have to plan five steps ahead rather than just three, the rewards are drawn from a Gaussian distribution rather than from a discrete uniform distribution, collecting information is three times as costly, and the cover story is different. As in the training task, more distal rewards had higher variance and therefore a backward planning strategy was still adaptive.

As shown in Fig. 3*B*, we found significant transfer effects from the relatively simple three-step training task to the more complex five-step transfer task (Kruskal–Wallis: H=13.02,P=0.001). Specifically, participants who had practiced with metacognitive feedback achieved an average relative test score of 74.6 points (95% CI [67.2, 81.4]) on the transfer task, significantly better than participants who had practiced with action feedback (57.0 points; 95% CI [49.0, 64.7], d=0.48,Z=3.14,P=0.002) or no feedback (57.5 points; 95% CI [48.8, 66.0], d=0.44,Z=2.91,P=0.004). This transfer effect was fully mediated by people learning to plan backward (*SI Appendix*, *SI Results*).

### Experiment 3: Retention.

Experiment 3 modified experiment 2 by adding a 24-h delay between the training task and the transfer task. We found that the transfer effect observed in experiment 2 was retained over time (H=28.66,P<0.001; Fig. 3*C*). Participants who had practiced with metacognitive feedback achieved a relative test score of 81.2 points (95% CI [74.9, 86.7]) on the delayed transfer task, significantly better than participants who had practiced with action feedback (49.0 points; 95% CI [39.6, 58.3], d=0.88,Z=5.08,P<0.001) or no feedback (57.6 points; 95% CI [48.1, 66.3], d=0.69,Z=4.04,P<0.001). This benefit was fully mediated by an increase in backward planning (*SI Appendix*, *SI Results*).

### Experiment 4: Metacognitive Feedback Is Also Effective in an Unstructured Environment.

The environments used in experiments 1 to 3 shared a simple structure that affords an intuitive strategy. To show that the effectiveness of our approach does not depend on this simplicity, we applied our method to an environment without any obvious structure. Importantly, we derived optimal feedback using exactly the same method, demonstrating the generality of our approach. In this new environment the rewards at all three levels are drawn from the same discrete uniform distribution with the possible values –10, –5, + 5, and + 10. The optimal strategy for this environment prioritizes collecting more information about the paths that appear most promising, prefers inspecting nodes that are informative about multiple paths, and uses a complex adaptive stopping rule (25).

**Fig. 4.**
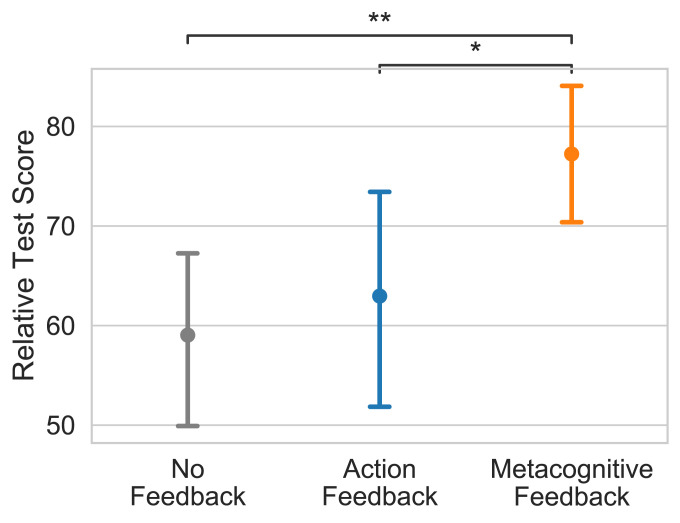
Metacognitive feedback improved people’s performance in an environment where the rewards are independently and identically distributed across all locations. *P < 0.05, **P < 0.01.

As illustrated in Fig. 4, metacognitive feedback was also effective in the unstructured environment (H=9.10,P=0.011). Participants who trained with the cognitive tutor achieved a relative test score of 77.2 points (95% CI [70.4, 83.8]), significantly better than participants who had practiced with action feedback (63.0 points; 95% CI [52.4, 73.5], d=0.42,Z=2.16,P=0.030) or without feedback (59.0 points; 95% CI [50.0, 67.7], d=0.61,Z=3.04,P=0.002). This improvement was accompanied by an increased probability of using a sophisticated planning strategy that is similar to the optimal one, from 0.6% in the control condition without feedback to 16.8% in the experimental condition with metacognitive feedback (U=1,136.5, *P* < 0.001). This strategy searches for a branch that starts with a positive outcome and then skips ahead to check the final outcomes along that branch, repeating this procedure until a path with positive initial and final outcomes is found. A more detailed analysis of how the tutor’s feedback affected people’s planning strategies in the unstructured environment is presented in *SI Appendix*.

### Experiment 5: Transfer to New Situations.

**Fig. 5.**
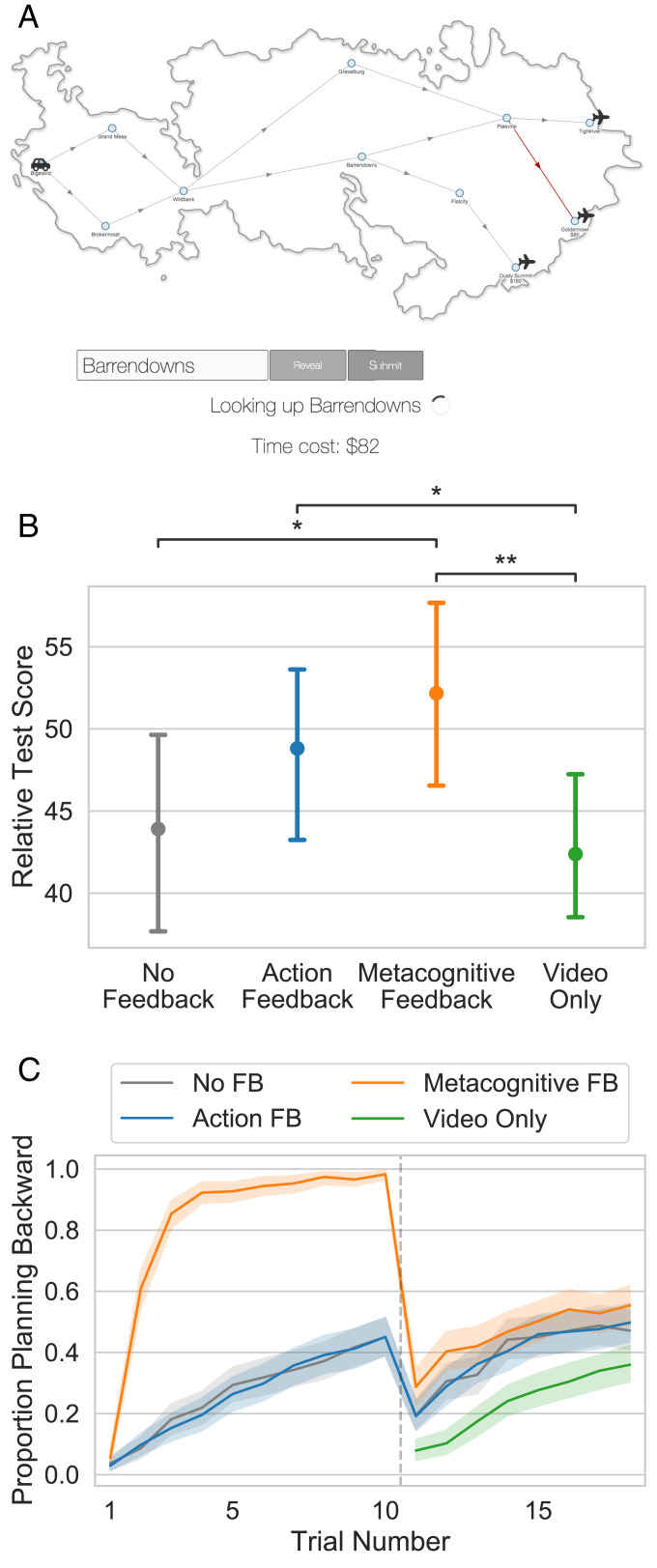
Far transfer. (*A*) The task environment shares the core property that rewards are more variable in distant states, but bears little resemblance to the training task. (*B*) Transfer performance in each condition. (*C*) Proportion of participants planning backward on each trial. The dashed line indicates the switch to the transfer task.

The goal of experiment 5 was to determine which benefits of training with a cognitive tutor transfer not only to larger environments within the same domain but also to other domains. To answer this question, we examined whether the benefits of practicing planning in the rather artificial Web of Cash task transfer to the more naturalistic task of planning an inexpensive road trip by using a search engine to look up hotel prices, namely the Road Trip paradigm illustrated in Fig. 5*A* (39). Critically, the road trip was required to end at a city with an airport, and the prices of hotels in these cities were highly variable; this makes the backward-planning strategy taught by our cognitive tutor highly adaptive for planning road trips in the transfer task.

In three conditions, participants practiced in the Web of Cash environment with metacognitive feedback, action feedback, or no feedback. To mimic a potential real-world application of our cognitive tutor, the training was followed by a series of questions that encouraged participants to reflect on what they learned and in which other situations it might be applicable. To investigate the extent to which the Web of Cash task itself produces transferable benefits, we added an additional control condition in which participants watched a video about if–then plans (40). In all four conditions, participants performed the Road Trip task immediately after completing the training phase. Because we expected the cross-domain transfer effects to be relatively small, we preregistered one-tailed tests for all our critical directional hypotheses.*

Performance on the transfer task differed significantly between the training conditions (H=8.87,P=0.031; Fig. 5*B*). Specifically, participants who trained in the Web of Cash task with metacognitive feedback performed significantly better on the transfer task (52.17 points; 95% CI [46.58, 57.67]) than participants who practiced without feedback (43.91 points; 95% CI [38.08, 49.78], d=0.18,Z=2.00,P=0.023) and those who watched a video about If–Then plans (42.38 points; 95% CI [37.40, 47.43], d=0.23,Z=2.56,P=0.005). They also performed slightly better than those who practiced with action feedback (48.81 points; 95% CI [43.26, 54.37]) but this difference was not statistically significant (d=0.08,Z=0.84,P=0.202).

The difference in overall performance was accompanied by a difference in the propensity to use backward planning strategies (H=37.00,P<0.001; Fig. 5*C*). As before, almost all participants receiving metacognitive feedback learned to use the adaptive backward planning strategy, compared to around half of the participants in the other training conditions. In all conditions, the rate of backward planning dropped dramatically on the first transfer trial. Nevertheless, participants in the metacognitive feedback condition checked the price of an airport hotel first on 46.3% of the transfer trials, significantly more often than the participants in each other condition (action feedback 39.4%, d=0.17,Z=1.81,P=0.035; no feedback 39.4%, d=0.17,Z=1.82,P=0.035; video 23.5%, d=0.61,Z=6.45,P<0.001). This increase in backward planning fully mediated the effect of training on test performance (average causal mediation effects 12.9 points, 95% CI [7.6, 18.1], *P* < 0.001; average direct effects –3.8 points, 95% CI [–8.0, 0.4], *P* = 0.082).

### Experiment 6: Mechanisms of Metacognitive Feedback.

The intelligent tutor’s metacognitive feedback has two components: a delay penalty and a message describing what the optimal heuristic would have done. The delay penalty serves as a negative reward that should drive the basic reinforcement learning mechanisms identified by recent models of metacognitive learning (10, 15, 28, 29). The message is a form of supervised learning signal that could be used by social learning mechanisms, such as imitation learning or reasoning about the tutor’s pedagogical goals (41, 42). To discern the contribution of these two components, we compared the effects of metacognitive feedback with versus without delay penalties and with versus without information about the optimal heuristic (*SI Appendix*, Fig. S10).

**Fig. 6.**
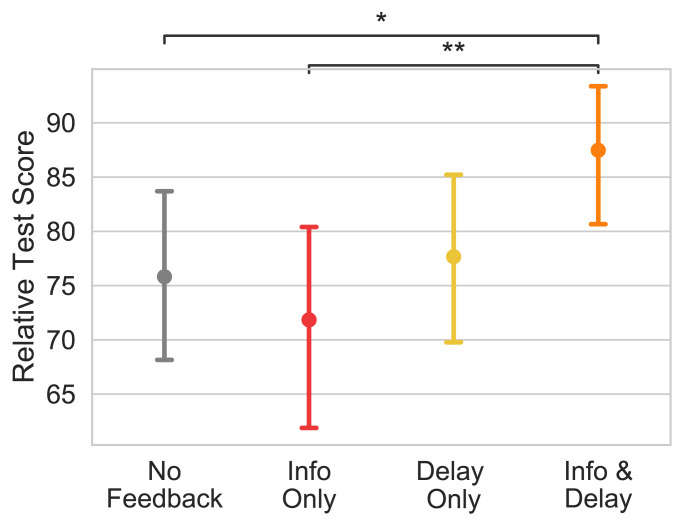
Test performance with different subsets of the two components of metacognitive feedback (delay penalties and information about the optimal heuristic).

As shown in Fig. 6, participants varied significantly in their performance based on which subset of optimal feedback elements they received (H=9.11,P=0.028). Consistent with our previous results, metacognitive feedback with both delay penalties and information about the optimal heuristic (87.5 points; 95% CI [80.6, 93.3]) significantly improved performance in the test block compared to practice without feedback (75.8 points; 95% CI [67.6, 83.2], d=0.32,Z=2.26,P=0.024). But neither delay penalties alone (77.7 points; 95% CI [69.6, 85.4], d=0.05,Z=0.33,P=0.743) nor information about the optimal heuristic alone (71.8 points; 95% CI [62.9, 80.2], d=−0.09,Z=−0.67,P=0.505) had a significant effect relative to no feedback. These results suggest that both components of our tutor’s metacognitive feedback are critical.

## Discussion

Decision-making skills are fundamental to the success of people, organizations, and society as a whole. To be able to make good decisions, we need clever decision strategies that direct our limited attention to the most important factors. Unfortunately, in many real-world environments the quality of the feedback people receive about their decisions is not good enough for them to discover such strategies on their own (13, 14, 16).

We developed an intelligent system that automatically discovers optimal decision strategies and teaches them to people by giving them metacognitive feedback while they are deciding what to do. The general approach starts from modeling the kinds of decision problems people face in the real world along with the constraints under which those decisions have to be made. The resulting formal model makes it possible to leverage artificial intelligence to derive an optimal decision strategy. To teach people this strategy, we then create a simulated decision environment in which people can safely and rapidly practice making those choices while an intelligent tutor provides immediate, precise, and accurate feedback on how they are making their decision. As described above, this feedback is designed to promote metacognitive reinforcement learning (10, 15, 28, 29).

We found that our intelligent tutor’s metacognitive feedback enabled people to rapidly discover effective decision strategies. Our training method outperformed two conventional approaches to cognitive training and improving human decision making (i.e., practice and performance feedback) and achieved promising transfer effects that were retained over time. Our cognitive tutor for decision making in situations where distant outcomes are more important than proximal outcomes enabled people to overcome their over-reliance on immediate rewards and to instead focus on the values of potential goals they could reach in multiple steps.

Our approach to designing intelligent cognitive tutors was successful in both a structured environment that affords a simple intuitive strategy (experiments 1 to 3) and an unstructured environment with a more complex optimal strategy (experiment 4). The results of experiment 5 suggested that people can transfer the strategies they learned from our cognitive tutor to more naturalistic tasks in different domains. Together, the findings from experiments 1 to 5 suggest that our intelligent tutor can help people learn how to plan better and make more far-sighted decisions. The findings of experiment 6 suggested that both the delay penalty and information provided by the tutor are important to its success, with the delay penalty playing an especially critical role. In an additional follow-up experiment (experiment 7; *SI Appendix*) we showed that people continue to use the strategy taught by the cognitive tutor even when it is not especially effective (nor particularly ineffective) in the new environment. This finding further supports the interpretation that the improvements in people’s decision making observed in experiments 1 to 5 were due to people learning a concrete planning strategy from the tutor’s feedback rather than due to people gaining insights into the structure of the environment. A further experiment (experiment 8; *SI Appendix*) suggested that although it is necessary that the training task is complex enough to capture the essential structure of the real-world environment, there are diminishing returns for increasing the complexity of the training task further.

The main contribution of this article is to lay the scientific and computational foundations for a principled approach to improving human decision making. The basic idea of this approach is to give people optimal metacognitive feedback on how they make decisions in the real world. To make this possible, we have laid down a theoretical foundation comprising a mathematical theory of optimal decision strategies, a conceptual theory of how people learn how to decide, and an automatic method for computing optimal metacognitive feedback. We have empirically validated this theoretical framework in a series of training experiments showing that giving metacognitive feedback is more effective at improving human decision making than giving performance feedback or letting people gain practical experience without feedback. These experiments support the efficacy of our general method for computing optimal metacognitive feedback.

The results of experiment 7 suggested that people find it challenging to discern situations in which the strategy taught by our intelligent cognitive tutor is beneficial from situations in which it is not. This highlights developing pedagogical interventions for helping people learn which kinds of situations the taught strategy is suitable for and how to recognize them as an important direction for future work. We believe that this is an important problem that should be solved before intelligent cognitive tutors of the kind introduced in this article are deployed to the real world.

The benefits of cognitive training are often limited to tasks that are very similar to the exercises that people practiced on (43, 44). This is known as the transfer problem. Indeed, in experiment 5, we found that the benefits of training with the tutor were substantially diminished in the far-transfer task. Moreover, the effect size estimate we obtained in experiment 5 is likely an upper bound on any transfer we can expect to see in the real world because the fact that both tasks were part of the same online experiment made it easier for participants to infer that the two tasks are related than it would have been in a real-world application.

To sidestep the far-transfer problem, future work could try to leverage automatically generated metacognitive feedback to train people on tasks that are similar or identical to those real-world tasks on which their performance shall be improved. Indeed, follow-up studies are beginning to demonstrate that our general method can be applied to real-world decisions that people make at their computers or smartphones. One concrete real-world application is training people to stay focused on the task they have chosen to work on. This can be done by creating an app that gives people feedback on how well the websites and programs they view on their computer match their intentions (45); that is, when people get distracted from a self-chosen task, they receive negative feedback and when they refocus on the task, they receive positive feedback. Another future application is to develop intelligent tutors that help people unlearn cognitive biases that lead to unfair discrimination. Since people’s cognitive biases are a direct consequence of the heuristics they use, teaching them to use heuristics that are adaptive for the specific decisions they face in their everyday life is a promising approach to helping people overcome systematic errors. Given that transfer is generally difficult to achieve, this should be pursued by augmenting specific real-life decision environments with metacognitive feedback. For instance, a program in which admission officers or recruiters evaluate applicants could be equipped with metacognitive feedback on which pieces of information they inspect first (e.g., gender versus qualifications) and which information they ignore. This real-world problem is an instance of multialternative, multiattribute decision making that can be formalized as a metalevel MDP (10, 46).

The metalevel MDP models of decision making in the real world will often be significantly larger than those we solved here. Therefore, computing the optimal metacognitive feedback for such real-world applications will require a machine-learning method that is more scalable than the backward induction algorithm we used here. In recent work, we have developed such methods and successfully applied them to larger metalevel MDPs (34, 46, 47).

Modeling real-world decision problems can be difficult because the structure of real-world problems is only partly known. To address this problem, our strategy discovery methods can be combined with Bayesian inference on the structure of the decision problem in a way that makes them more robust to model misspecification (48).

One limitation of our proof of concept is that participants might perform additional unobserved planning operations for which they receive no feedback. This does not take away from the value of the feedback that our tutor gives on the planning operations that we do observe, but it does suggest that the effectiveness of our cognitive tutors can be improved even further. One simple extension that would allow our cognitive tutor to give feedback on an even larger proportion of people’s planning operations would be to reveal each piece of information only briefly. As a result people might then click on the same piece of information multiple times when it is used by multiple planning operations. Future work can also use eye tracking to obtain an even more accurate measure of people’s planning operations. Another limitation is that we approximated the cost of planning by the fees that participants paid to collect information. Future work should obtain more realistic measures of the cost of planning by measuring the mental effort and time that it takes people to process the acquired information. Measuring and modeling people’s planning operations and their cognitive costs more accurately may lead to cognitive tutors that are even more effective. Another important direction for future work is the development of tasks that make planning operations measurable without reducing their associated computational costs.

How much a person benefits from receiving metacognitive feedback can be limited by how the person represents the decision problem. This is a concern because the way in which people represent a task determines which features they attend to, to learn which actions will be rewarded (49). Therefore, the benefit that learners can derive from metacognitive feedback is limited by their mental representation of the problem they are trying to solve. However, this does not mean that a person who does not already represent the problem in the best possible way cannot learn an optimal strategy. To the contrary, people can flexibly improve their representations (50). In particular, they can learn representations that enable them to predict which actions will be rewarded (51–53). This likely also applies to planning operations. We therefore expect that metacognitive feedback can also help people learn more adaptive problem representations that make it easier for them to discover effective decision strategies. Testing this prediction is an interesting direction for future research. While reinforcement informs representation learning, there are also many other representation learning mechanisms, such as categorization, that shape how people represent decision problems (49). Therefore, future work should investigate how our intelligent tutors can be extended to more effectively help people (learn to) represent decision problems in ways that make it easy for them to discover and apply resource-rational heuristics. One simple approach could be to highlight the aspects of the problem’s structure that the optimal strategy relies on.

To scale up our approach to improving human decision making to more complex real-world scenarios, it will be important to ensure that people can understand why the feedback makes sense and what the tutor is trying to teach them. To achieve this level of interpretability, we are currently developing automatic methods for generating human-interpretable descriptions of the optimal decision strategies taught by our intelligent tutors (54). Furthermore, the tutor’s metacognitive feedback can be enhanced with text that explains why it would have been better to perform an alternative operation. Such explanations can be given automatically by comparing the common features of optimal operations (e.g., “inspects a final outcome”) to the corresponding features of the chosen operation (e.g., “inspects an immediate outcome”). We believe that augmenting our intelligent tutors with interpretable descriptions of the strategies that are being taught and explanations of why an alternative operation would have been better will enable people to learn from the tutor’s feedback even when the problem is more complex than the simple tasks we used to provide a proof of concept.

The findings presented in this article provide a proof of concept for a general method for improving people’s decision-making abilities. Our computational framework for discovering and teaching resource-rational cognitive strategies could lead to a principled approach to improving the human mind. Future work will apply our approach to increasingly more realistic scenarios, such as planning how to reach a project milestone and deciding which company to invest in, and address the transfer problem and other challenges associated with improving decision making in the real world.

## Materials and Methods

Our data, the analysis code, and a demonstration of the experimental paradigm are available at https://github.com/fredcallaway/ai-for-improving-human-planning. The experiments reported in this article were approved by the institutional review board of the University of California, Berkeley, under Institutional Review Board (IRB) Protocol 2015-05-755 (“Cognitive Research Using Amazon Mechanical Turk”); the institutional review board of Princeton University under Protocol 10859 (“Computational Cognitive Science”); and the Independent Ethics Committee of the University of Tübingen under IRB Protocol 667/2018BO2 (“Online-Experimente über das Erlernen von Entscheidungsstrategien”). All participants of all experiments gave informed consent in advance.

To quantify participants’ task performance in a way that is interpretable and comparable across all of our experiments, we measured their score relative to the expected scores of an (approximately) optimal strategy and guessing randomly. Given the raw score *s* achieved on a given trial the relative score is given by

[2] $$\text{relative-score}(s)=100\cdot \frac{s-{\bar{s}}_{\text{rand}}}{{\bar{s}}_{\text{opt}}-{\bar{s}}_{\text{rand}}},$$

where ${\bar{s}}_{\text{opt}}$ is an (approximate) upper bound on possible average score and ${\bar{s}}_{\text{rand}}$ is the expected score achieved by randomly selecting paths without doing any planning. We set the upper bound to the performance of the optimal policy when this was possible to compute. For the large near-transfer environment (which is too large to solve exactly) and for the far-transfer environments (where the rewards are not independently identically distributed), we instead approximated this upper bound by the performance of a goal-setting strategy, which approximates the strategy taught by the tutor. This strategy checks terminal states until finding one with reward above a threshold and then selects a path to that state or, if no such state is found, the terminal state with maximal reward. The threshold was optimized to maximize the strategy’s performance.

All permutation tests were performed using the R package “coin,” using the default asymptotic approximation method (37). The causal mediation analysis was performed using the R package “mediation” (38). The mediator “backward planning” was operationalized by whether or not the participants’ first click fell on one of the nodes they would reach after their third and final move. Both analyses were performed at the participant level; we therefore averaged the values of the mediator (backward planning) and the dependent variable (“relative score”) across the 20 trials of the test block. Confidence intervals were computed (also at the participant level) by Monte Carlo permutation with 10,000 samples.

### Experiment 1.

We recruited 151 participants on Amazon Mechanical Turk (average age, 34.5 y; range, 18 to 72 y; 72 females). For all experiments, balanced condition assignment and repeat-participant exclusion were performed using psiTurk (55). Each participant was assigned to receive metacognitive feedback (50 participants), action feedback (50 participants), or no feedback (51 participants) during the training block. The experiment comprised instructions, a training block, a test block, and an exit survey. The training block comprised 10 trials, and the test block comprised 20 trials. The exit survey asked participants about what they had learned, their age, and their gender identity.

Each trial presented participants with an instance of the three-step planning problem described above (Fig. 1*A*). The key structure of this problem is that the range of possible rewards is smallest in the first step, larger in the second step, and largest in the third step. To operationalize the cost of planning, we charged participants one virtual dollar per click. To simplify the implementation of metacognitive feedback, we required that all clicks be made before the first move (note that it is never optimal to click after moving because the state transitions are deterministic). To eliminate the time cost of engaging in planning compared to speeding through the experiment, participants who spent less than 7 s on planning (e.g., only 3 s) had to wait for the remaining time after executing their moves (e.g., for 4 s). In the test block, participants started with an endowment of 50 virtual dollars and earned a bonus of $0.01 for every $5 they made in the game.

In both feedback conditions, the feedback consisted of a delay penalty (negative reinforcement) as well as a message indicating what the best thing to do was. In the metacognitive feedback condition, feedback was given after each planning operation, including both clicks and the decision to stop planning and move the spider. The delay penalty was 2+a·loss(b,c) seconds (Eq. **1**) if the participant made an error or 0 seconds if the participant’s planning operation was optimal. We chose the value of the scaling factor *a* so that the delay for acting without planning was 42 s (*SI Appendix*, *SI Methods*). If the optimal operation was to click but the participant did not make an optimal click, the optimal nodes to click were highlighted and a message was displayed: “You should have inspected one of the highlighted nodes.” If the optimal operation was to move but the participant clicked, the message read “You shouldn’t have inspected any more nodes.” In the action feedback condition, the tutor gave feedback on participants’ first moves but not on the their planning operations (clicks). The delay penalty was determined using the same equation, but replacing the metalevel Q function with the “task-level” Q function; that is, the loss function compares the total reward one would receive from taking the optimal path following that initial action to the maximal total reward one could obtain by starting with the best possible move. If the participant chose the wrong direction, the message read “You should have moved left/up/right” depending on which direction would have been optimal given full information.

### Experiment 2.

The methods of experiment 2 were the same as in experiment 1 unless stated otherwise. We recruited 297 participants on Prolific (average age, 32.2 y; range, 18 to 71 y; 158 females). We excluded 25 participants who reported possible participation in a previous version of the experiment (e.g., on Mechanical Turk or with a different Prolific account).

Each participant was assigned to receive metacognitive feedback (99 participants), action feedback (100 participants), or no feedback (98 participants).

The transfer task illustrated in Fig. 3*A* was a five-step sequential decision problem. It was framed as routing an airplane across a network of airports. The rewards of nodes at step i∈{1,2,3,4} were drawn from normal distributions with mean zero and SD $\sigma_{i}=2^{i-1}$, and for the rewards at the last step (*i* = 5) the SD was $\sigma_{5}=2^{5}$. This reward structure was chosen to ensure that the backward-planning heuristic identified as optimal for the smaller training environment was near optimal for the transfer environment as well. Unlike in the training task, the cost of planning was $3 per click.

### Experiment 3.

This experiment employed the training block and the transfer task from the transfer experiment (experiment 2) with an added a 24-h delay between the training block and the transfer task.

We recruited a total of 297 participants on Prolific (average age, 31.9 y; range, 18 to 76 y; 119 females). We excluded 18 participants who reported possible participation in a previous version of the experiment. Each participant was assigned to the experimental condition that trained with the intelligent tutor (101 participants), the control condition that practiced with action feedback (99 participants), or the control condition that practiced without feedback (97 participants).

The 24-h delay was accomplished by splitting the experiment into two stages, the second of which could only be begun 24 h after beginning the first stage. The first stage comprised instructions and a training block. The second stage comprised instructions reminding participants how the game works, the transfer block where participants were posed 20 five-step planning problems (Fig. 3*A*), and the closing survey used in experiments 1 and 2. About 83.5% of the participants of stage 1 returned to stage 2 (i.e., 248 of 297). The proportion of participants who dropped out after the first stage did not differ substantially between the three conditions (metacognitive feedback, 15.8%; action feedback, 16.2%; no feedback, 17.5%).

### Experiment 4.

We recruited 179 participants on Prolific (average age, 32.4 y; range, 18 to 74 y; 87 females). We excluded 16 participants who reported possible participation in a previous version of the experiment. Sixty participants were assigned to the experimental condition with metacognitive feedback, 58 were assigned to the control condition with action feedback, and 61 participants were assigned to the control condition without feedback.

The task environment differed from the one used in experiment 1 in that the rewards at all three levels were drawn from a discrete uniform distribution over the possible rewards –10, –5, + 5, and + 10. In the test block the task environment was the same as in the training block and all three experimental groups solved 20 planning problems without any feedback. The penalty delays were calculated such that the metacognitive feedback for acting without planning was the same as in the cognitive tutor used in experiments 1 to 3.

### Experiment 5.

We recruited 1,380 participants on Prolific (average age, 25.23 y; range, 18.0 to 57.0 y; 459 females). We excluded 112 participants who reported possible participation in a previous version of the experiment.

The experiment comprised general instructions, a training block, questions designed to promote transfer (transfer prompts), and a test block in which participants were evaluated on a new transfer task. The four conditions of the experiment differed only in the training block. In this training block, three groups practiced planning in 10 trials of the Web of Cash game with optimal metacognitive feedback (233 participants), action feedback (235 participants), or no feedback (242 participants). An additional control group (253 participants) watched a video about If–Then plans (40) instead of practicing in the Web of Cash environment.

The transfer prompts told participants that the Web of Cash game is a metaphor for life and asked them to articulate the lesson they had learned, think about a situation in which it might be applicable, and describe how it could be applied to efficiently plan a road trip (see *SI Appendix*, *SI Methods* for more detail).

In the test block, all participants completed eight trials of a modified version of the Road Trip paradigm introduced in ref. 39 (Fig. 5*A*). In this task, participants play the role of a travel agent tasked to plan an inexpensive road trip from the client’s current location to any city with an airport. To make an informed recommendation, they can look up the price of each city’s most affordable hotel by typing the city’s name into a search engine. The participant has to be economical with time because the travel agent is working from a very expensive internet cafe, which charges them $0.25/s, and the search engine takes 4 s to find the cheapest rate in the queried city. In each round the travel agent starts with a budget of $800 for the trip and the agent’s internet research and the participant earned a bonus of $0.01 for every $2 left from that budget. Unbeknownst to the participants, the hotels in cities with airports have a much wider range of possible prices ($100, $320, $350, or $380) than the hotels in other cities ($130, $135, $140, or $145). Furthermore, exactly one of the airport hotels always had the lowest price of $100, making it possible to always find an inexpensive route.

The transfer task differed from the training task on several important dimensions. First, the transfer task is much more naturalistic than the training task: It mimics the real-life challenge of planning a road trip, it captures that researching prices is effortful and time consuming, and it captures that each destination can be reached via multiple routes. Second, while the training task asks people to maximize profits, the transfer task asks them to minimize costs. Third, unlike the training task, the transfer task had to be performed under time pressure. Last but not least, the transfer task has a very different user interface than the training task (cf. Fig. 5*A* vs. Fig. 1*A*). In particular, participants obtained information by typing text into a search box (vs. clicking) and they selected their route by clicking on the roads between cities (vs. arrow keys).

### Experiment 6.

Experiment 6 disentangled the effects of reinforcement versus information about the optimal heuristic using a 2 × 2 factorial design with the factors delay penalties (present vs. absent) and information about what the optimal heuristic would have done (present vs. absent). We recruited 417 participants on Amazon Mechanical Turk (average age, 36.0 y; range, 18 to 87 y; 204 females). Participants were assigned to receive no feedback (104 participants), only information (104 participants), only delay penalties (104 participants), or the full feedback with both information and delay penalties (105 participants).

Following the instructions, participants completed 10 training trials and 15 test trials. Finally, each participant completed the exit survey described above. During the training trials each of the four conditions received a different type of feedback. Depending on the experimental condition, the feedback included a delay penalty, information about what the optimal heuristic would have done, both, or neither one. During the test trials, none of the groups received feedback. Both training and test trials used the three-step planning task from experiment 1.

In contrast to previous experiments, all clicks in all conditions were followed by an unconditional delay of 1 s before revealing the reward at the clicked state. This unconditional delay afforded enough time to show the information feedback when applicable, without introducing any behavior-dependent delays or unintended differences between conditions. Participants in the conditions with delay penalties received an additional delay penalty whose duration was proportional to how much worse their planning operation was than the optimal one. During the delay penalty, participants were shown the message “Delay penalty for poor planning: *x* seconds” (where *x* is the duration of the delay). When the participant’s planning operation was optimal, the message read “Good job!” and there was no additional delay. In the conditions with information, after a suboptimal planning operation, a visual illustration of what the optimal heuristic would have done differently (*SI Appendix*, Fig. S10) was displayed during the entire delay period, which was at least 1 s and longer in the condition with delay penalties. Finally, to roughly match the amount of time spent in the experiment, in conditions with delay penalties, all training trials were followed by an 11-s delay during which we told participants that the next trial was being prepared. In conditions with delay penalties, this posttrial delay was 1 s. The difference of 10 s was chosen to offset the average total delay penalty per trial in experiment 1.

## Supplementary Material

Supplementary File

## Data Availability

Anonymized datasets have been deposited in GitHub (https://github.com/fredcallaway/ai-for-improving-human-planning).

## Footnotes

*https://aspredicted.org/as5ib.pdf.
