# Supplementary material for: Leveraging artificial intelligence to improve people’s planning strategies
Source: Proc Natl Acad Sci U S A. 2022 Mar 16;119(12):e2117432119. doi: 10.1073/pnas.2117432119 (PMC8944825; doi:10.1073/pnas.2117432119)
Supplement: Supplementary File [file pnas.2117432119.sapp.pdf]

Supporting Information

Callaway et al. 10.1073/pnas.XXXXXXXXXX

SI Results

Figure S1 shows the full distribution of all individual participants' average scores for each condition of each experiment reported in the main text.

Experiment 1.

A closer look at the effect of metacognitive feedback on strategy learning Figure S2 visualizes our finding that participants who received metacognitive feedback were more likely to start by inspecting one of the potential final locations (Step 3) and less likely to start by inspecting one of the immediate (Step 1) or intermediate outcomes (Steps 2).

To gain a closer understanding of how people's planning strategies changed during learning depending on whether they received feedback and which kind of feedback they received we analyzed the process-tracing data from Experiment 1 with the computational microscope developed by [1].

Inspecting people's decision strategies in the control condition revealed three short-sighted decision strategies: i) a myopic satisficing strategy that inspects the immediate outcomes of alternative actions until it encounters a positive outcome and then immediately chooses the corresponding action, ii) a myopic maximizing strategy that inspects each action's immediate outcomes and then chooses the action with the best immediate outcome, and iii) an overly frugal myopic strategy that inspects only a single immediate outcome and nothing else. In the control condition, 43.1% of the participants used one of these short-sighted strategies in their first trial; suggesting that more than one third of their decisions were overly swayed by immediate outcomes and ignored the more important long-term consequences. After an initial drop to about 16% within the first seven trials, the decrease in people's reliance on short-sighted decision strategies slowed down and 7.8% of the participants continued to use short-sighted strategies after 30 trials of training (see Figure S3). Encouragingly, as Figure S3 shows, optimal metacognitive feedback significantly accelerated the extinction of short-sighted decision strategies and reduced their prevalence to almost 0% within 7 trials; and the average frequency of short-sighted strategies in the test block was significantly lower for the people who had received metacognitive feedback than for people who had received no feedback (0.0% vs. 10.5%, $\chi^2(1) = 108.7$, $p < .0001$) and people who had received action feedback (9.5%, $\chi^2(1) = 111.0$, $p < .0001$). Furthermore, we found that our cognitive tutor enabled a larger proportion of people to transition to the optimal goal-setting than practice with action feedback (74.0% vs. 42.0%, $\chi^2(1) = 10.51$, $p = .0012$). In the control condition the frequency of the optimal strategy was lower than in the experimental condition with metacognitive feedback (56.9% vs. 74.0%) but this difference was not statistically significant ($\chi^2(1) = 3.27, p = .0700$).

Analyzing the effect of the metacognitive feedback on the frequencies with which people moved from one strategy to another suggested that optimal metacognitive feedback significantly increased the probability with which people moved away from choosing impulsively (Strategy 30) in [2] and eight other strategies (i.e Strategies 11, 16, 22, 31, 49, 53, 56, 65). Metacognitive feedback also increased the probability that people would stick with the optimal strategy (i.e Strategy 21) and three other high-performing strategies (i.e., Strategies $24, 57, 58$ from [2]; all $p < \alpha_{\text{Sidak}} = 1.84 \cdot 10^{-4}$).

Experiment 2.

Mediation analysis We found that people's people's propensity to look at potential final outcomes first fully mediated the effect of feedback on people's performance on the transfer task (average causal mediation effects: 17.8 points, 95% CI: [8.2, 27.0], $p < .001$; average direct effects: -0.5 points, 95% CI: [-7.2, 5.8], $p = .872$).

The primary mechanism by which metacognitive feedback improved performance on the transfer task was to encourage backward planning. Metacognitive feedback significantly increased participants' propensity to plan backward. Specifically, metacognitive feedback made participants plan backwards on 86.2% of trials compared to 64.5% in the No Feedback group. Using a backward planning strategy, in turn, increased participants' average scores by 81.7 points. The increase in participants' propensity to start by inspecting potential final outcomes emerged gradually over time and was boosted by metacognitive feedback (see Figure S4A).

Experiment 3.

Mediation analysis We found that the people's increasing propensity for backward planning fully mediated the effect of feedback on people's performance on the delayed transfer task (average causal mediation effects: 20.0 points, 95% CI: [10.3, 29.2], $p < .001$; average direct effects: 4.0 points, 95% CI: [-4.1, 12.0], $p = .342$).

Metacognitive feedback significantly increased participants' propensity to plan backward in the delayed transfer task. Specifically, metacognitive feedback made participants plan backwards on 93.6% of trials compared to 67.5% in the No Feedback group. Planning backward, in turn, increased participants' average scores by 78.8 point. The increase in participants' propensity to start by inspecting potential final outcomes emerged gradually over time and was boosted by metacognitive feedback (see Figure S4B).

Experiment 4. To investigate what participants learned from the metacognitive feedback, we applied a recently developed computational method for inferring people's planning strategies from the clicks they make in the Mouselab MDP paradigm [2] to the data from Experiment 4. We used this method to compare how often people used different planning strategies in the test block between the control group and the experimental group which had practiced with metacognitive feedback. To do this, we performed one pairwise comparison between the experimental group which had received metacognitive feedback and the control group who received no feedback for each strategy whose frequency was at least 3% in at least one of these two conditions ($\alpha_{\text{Bonferroni}} = 0.0033$). Table S1 summarizes

DRAFT

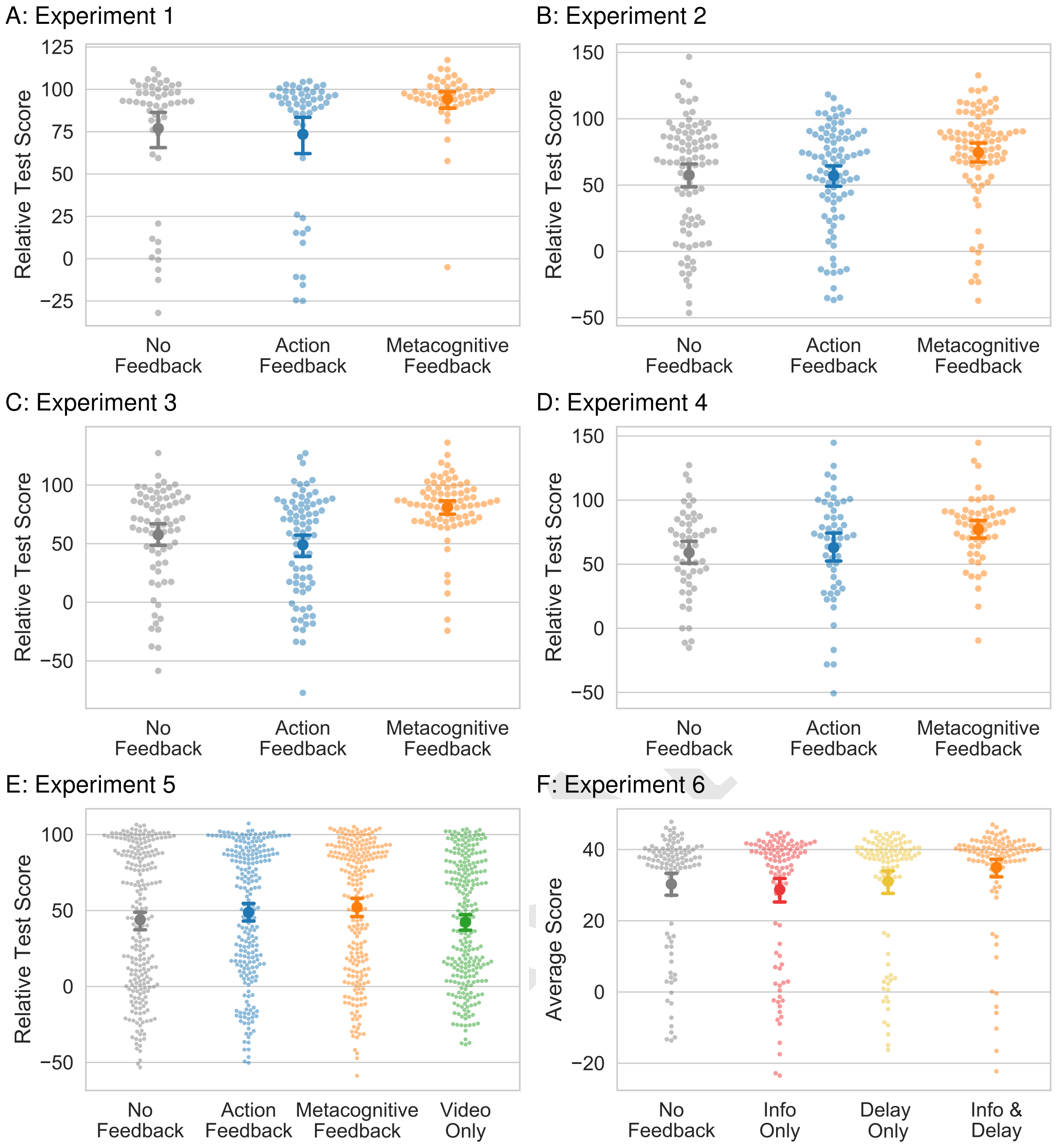

Fig. S1. Distribution of scores across participants in each experiment. The error bars convey 95% confidence intervals produced by 1000 bootstrap samples.

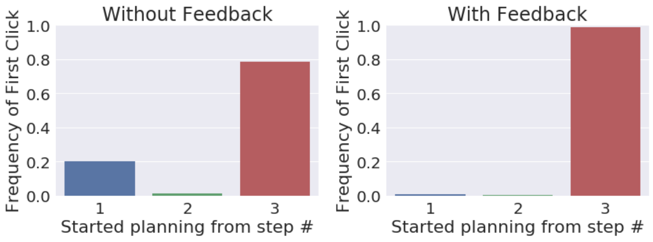

Fig. S2. Practice with the cognitive tutor promoted backward planning.

the results. These results show that metacognitive feedback led to an increase in the frequency of an adaptive forward-planning strategy similar to best-first search (Strategy 59; see Table S1). This increase was accompanied by a decrease in the frequencies of four less adaptive strategies: two strategies that inspect only immediate outcomes, a strategy that plans backward from final outcomes, and a less effective variant of the strategy whose frequency increased.

Experiment 5. For the far-transfer experiment, we preregistered several additional analyses that we ultimately removed from the main text for reasons of space. Note also, that we deviated from the preregistration by adopting a reviewer's

	Description	Change in Freq.	U	p
Strategy 59	Inspect the first outcome on some path. If it is positive, then inspect the final outcomes on that path. Else inspect another unknown first outcome. Repeat this procedure until you uncover a positive final outcome or there are no immediate outcomes left to inspect. If no positive immediate outcome was found, then inspect the final outcomes until you find a positive final outcome.	0.6% → 16.8%	1136.5	.0002
Strategy 8	Inspect all immediate outcomes except for one.	17.0%→0.4%	1254.0	.0027
Strategy 51	Explore immediate outcomes until you uncover a positive outcome or all immediate outcomes have been inspected(Step 1). If you uncovered a positive immediate outcome, then inspect the final outcomes following the positive immediate outcome. If one of them is positive, then stop, else continue with Step 1.	4.3%→0.0%	0.0	$<$ .0001
Strategy 53	Inspect immediate outcomes until you uncover a positive immediate outcome.	3.8%→0.0%	0.0	$<$ .0001
Strategy 75	Inspect the final outcomes until you uncover the largest possible value (Step 1). Then inspect the locations on the path from the initial location to that final outcome (Step 2). Then continue from Step 1 unless all final outcomes have already been inspected.	3.8%→0.0%	0.0	$<$ .0001

Table S1. Effect of metacognitive feedback on how often participants used different planning strategies in the test block of Experiment 4. Strategies whose frequencies significantly increased are shown at the top. Strategies whose frequencies significantly decreased are shown at the bottom. To be included in this table the change in the frequency had to be at least 3% and be statistically significant.

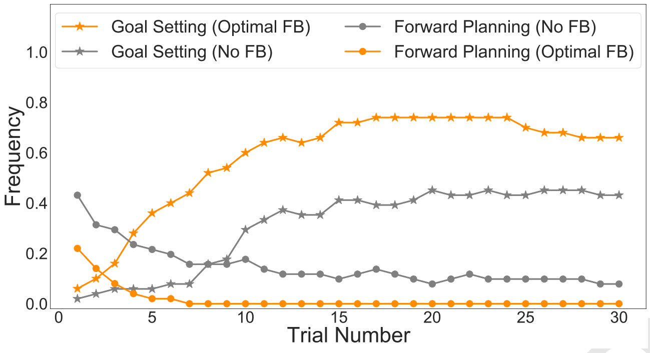

Fig. S3. Prevalence of short-sighted versus far-sighted planning strategies for participants practicing with versus without out cognitive tutor.

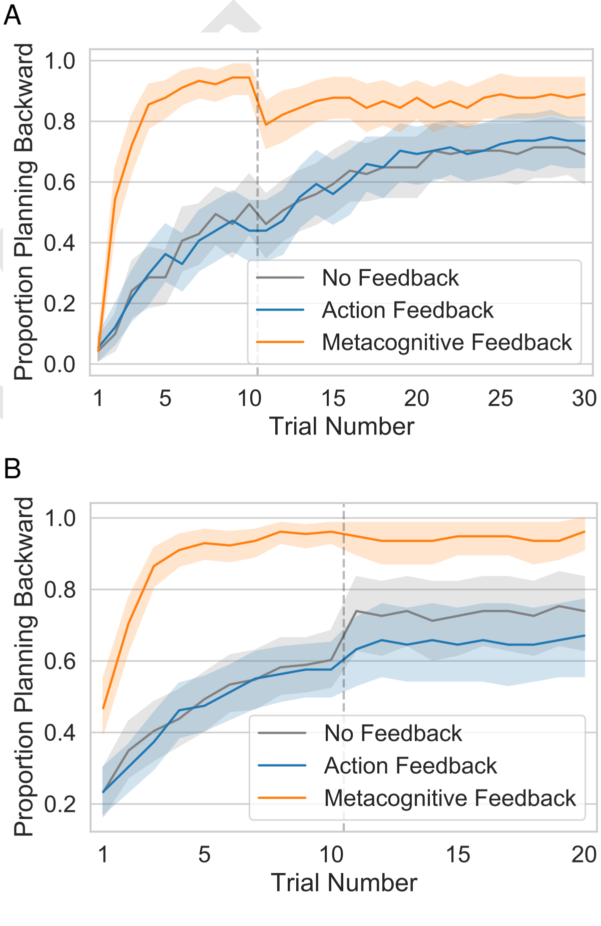

Fig. S4. Temporal evolution of the prevalence of backward planning in Experiments 2 and 3. Here, and in all future plots, the shaded regions indicate 95% confidence intervals.

suggestion to analyze the relative score instead of the bonus. Both analyses yielded very similar results with identical conclusions. The results were not exactly the same because the normalization is applied separately for each map, effectively reducing some item-related variance.

A Kruskal-Wallis ANOVA revealed a significant overall effect of training on participant bonuses ($H = 8.76, p = .033$). There was no statistically significant difference between metacognitive feedback versus action feedback ($d = 0.07, Z = 0.74, p = .230$). But there was a statistically significant benefit of metacognitive feedback over no feedback ($d = 0.18, Z = 1.97, p = .024$) and watching a video about If-Then plans ($d = 0.23, Z = 2.53, p = .006$). There was a statistically significant benefit of practicing with action feedback over watching a video about If-Then plans ($d = 0.16, Z = 1.76, p = .039$) but no such benefit from practicing without feedback ($d = 0.04, Z = 0.41, p = .341$).

A Kruskal-Wallis ANOVA did not reveal a significant overall effect of training on the cost of the routes participants selected ($H = 6.81, p = .078$). Note that this measure differs from total score by not including the time cost incurred while planning. As pre-registered, we nevertheless conducted all critical pairwise tests. There was no statistically significant difference between metacognitive feedback versus action feedback ($d = -0.08, Z = -0.85, p = .199$). But there was a

statistically significant benefit of metacognitive feedback over no feedback ($d = -0.20, Z = -2.16, p = .015$) and watching a video about If-Then plans ($d = -0.21, Z = -2.28, p = .011$). Finally, there was no statistically significant difference between watching a video about If-Then plans and action feedback ($d = -0.13, Z = -1.38, p = .083$) or no feedback ($d = 0.00, Z = 0.03, p = .512$).

A Kruskal-Wallis ANOVA did not reveal a significant overall effect of training on the probability that participants ever check the price of an airport hotel ($H = 4.26, p = .235$). As preregistered, we nevertheless conducted all critical pairwise tests. The difference between metacognitive feedback and watching a video about If-Then plans was statistically significant ($d = 0.17, Z = 1.83, p = .033$) but all other differences were not statistically significant (metacognitive feedback vs. action feedback: $d = 0.06, Z = 0.61, p = .271$; metacognitive feedback vs. no feedback: $d = 0.14, Z = 1.57, p = .059$; action feedback vs. video: $d = 0.11, Z = 1.21, p = .112$; no feedback vs. video: $d = 0.02, Z = 0.23, p = .410$).

Experiment 7: The tutor influences people's strategy more than the environment. In Experiments 1-6 the strategy taught by the cognitive tutor in the training block was always adaptive for the environment used in the test block. Experiment 7 investigated whether and to which extent people would continue to use the strategy taught by the intelligent tutor even when it is no longer adaptive in the environment used in the test block. For this purpose, the experimental group was taught the optimal strategy for the environment where distant outcomes are more important than immediate ones in the training block and tested in an environment where all locations are equally important. Their propensity to continue using the taught strategy was compared to a control group where the test environment was identical to the training environment.

As shown in Figure S5A, we found that most people continued to use the strategy taught by the cognitive tutor even when it was not especially well-suited to the environment of the transfer task. That is, over the course of the 20 transfer trials all but 27.5% of participants in the test group continued to use the backward planning strategy, when the range of possible rewards was the same in all three steps. Although high, this proportion was significantly lower than in the control condition where participants remained in the training environment (5.9%, $\chi^2(1) = 11, p < .001$). The difference between these two conditions became even clearer when we removed participants who did not plan at all ($\chi^2(1) = 243, p < .001$; see Figure S5B).

Experiment 8: Diminishing returns for making the training task increasingly more complex. Experiment 8 investigated whether the complexity of the training task impacts performance on the testing task (see Figure S11). We varied the complexity of the training task on which people practiced with the cognitive tutor to be either *minimal*, *easy*, or *difficult*, while holding the transfer task constant (see Figure S11). The *difficult* version was identical to the cognitive tutor tested in Experiments 1-3 where people have to choose a sequence of three actions; in the easy version of the training task, people had to choose a sequence of two actions; and in the minimal version of the task people chose a single action. Imporantly, both the easy and difficult conditions had higher variance for the final-step rewards than the initial-step rewards. This was

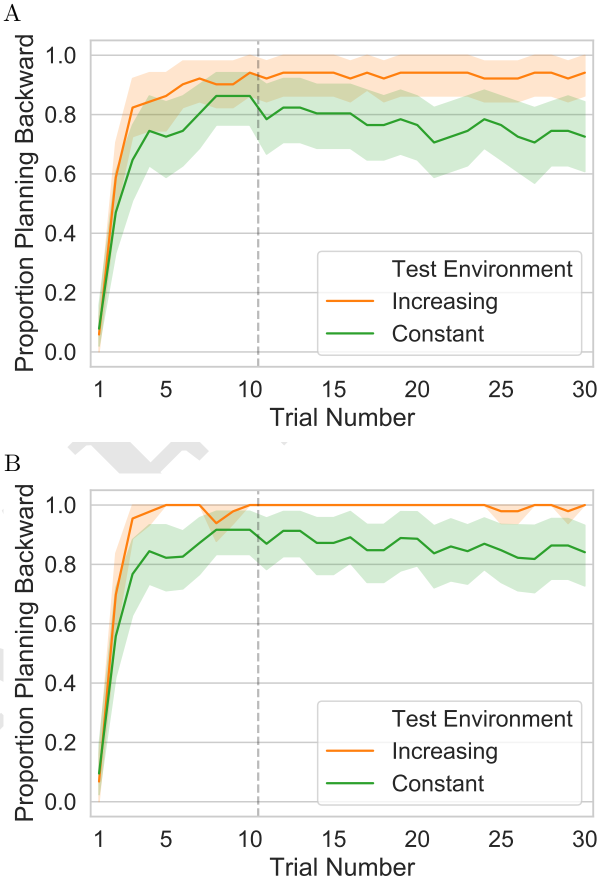

Fig. S5. Proportion of participants who used the backward planning strategy in Experiment 7 by trial number and experimental condition. Panel A shows the learning curve for all participants. Panel B shows the learning curve for only those participants who clicked at least once on the corresponding trial.

not possible for the minimal version because there was only one step. Following the training, all three groups were tested on the 5-step transfer task introduced in Experiment 2, but with a \$1 click cost instead of \$3.

The complexity of the training task used by the cognitive tutor (see Figure S11) had a significant effect on people's performance on the transfer task. Figure S6A shows the average scores for participants during training and testing, in each of the conditions of training complexity. Training complexity had a significant effect on performance ($H = 14.807, p < .001$). The differences between the three conditions were most pronounced in the beginning of the transfer block. In the first five trials of the transfer block, participants trained on the simple task (63.69 points; 95% CI: [54.76, 72.21]) performed significantly better than participants trained on the minimal task (49.10 points; 95% CI: [39.88, 57.93]; $d = 0.83, Z = 4.29, p < .001$). However, even in the first five trials, participants trained on the difficult task (67.08 points; 95% CI: [56.11, 76.97]) did not perform significantly better than participants trained on the simple task (49.10 points; 95% CI: [39.88, 57.93]; $d = 0.04, Z = 0.21, p = .831$). The additional performance increase attained by adding a third step to the training task was significantly smaller than the increase achieved by adding the second step ($p = .024$). This finding suggests that there are diminishing returns for increasing the complexity of the cognitive tutor's training task after it already captures the essential structure of the environment in which people's performance is to be improved.

Across the subsequent trials of the transfer block the differences between the conditions diminished but participants' performance still followed the same pattern. The average performance in the transfer block was significantly higher for participants who had been trained on the simple task (63.69 points; 95% CI: [54.32, 72.59]) than for participants who had been trained on the minimal task (49.10 points; 95% CI: [39.77, 58.28]; $d = 0.40, Z = 2.18, p = .029$). This finding suggests that it is critical that the training environment is complex enough to capture the essential structure of the transfer task. Although average performance on the transfer block was even higher for participants trained on the difficult task (67.08 points; 95% CI: [56.51, 76.94]), this improvement over training on the simple task was not statistically significant ($d = 0.09, Z = 0.48, p = .628$).

Participants' propensities to use the backward planning strategy that is optimal for the transfer environment mimicked these findings (see Figure S6B). The data shown in Figure S6B suggests that the effect of the training task's complexity on participants' performance on the transfer task was mediated by their propensity to use a strategy that is adapted to the structure of the transfer environment (i.e., planning backward). Participants who had been trained in the difficult task were significantly more likely to use the backward planning strategy in the first five trials of the transfer task than participants who had been trained on the minimal task (72.3% vs. 23.8%; $d = 1.27, Z = 6.01, p < .001$) and participants who had been trained on the simple task (72.3% vs. 54.2%; $d = 0.43, Z = 2.34, p = .019$. Furthermore, participants who had been trained in the simple task were significantly more likely to use the backward planning strategy in the first five trials of the transfer task than participants who had been trained on the minimal task (54.2% vs. 23.8%, $d = 0.81, Z = 4.19, p < .001$). As shown in Figure S6B, the differences between the conditions decreased over time as participants gradually adjusted to the environment of the transfer task. Across all 20 trials of the transfer block the proportions of backward planning were 37.1%, 63.0%, and 74.4% for the minimal, simple, and difficult task, respectively. The differences between the minimal task and two more complex tasks were statistically significant across the entire test block ($d = 0.81, Z = 4.19, p < .001$ and $d = 1.27, Z = 6.01, p < .001$, respectively). However, the difference between the simple task and the difficult task was not significant at the $\alpha = .05$ level when all 20 trials of the transfer task were considered ($d = 0.43, Z = 2.34, p = .019$).

Overall, the findings of Experiment 8 suggest that while it is critical that the training task is complex enough to capture the essential structure of the real-world environment, the returns for additional increases in complexity diminish rather quickly.

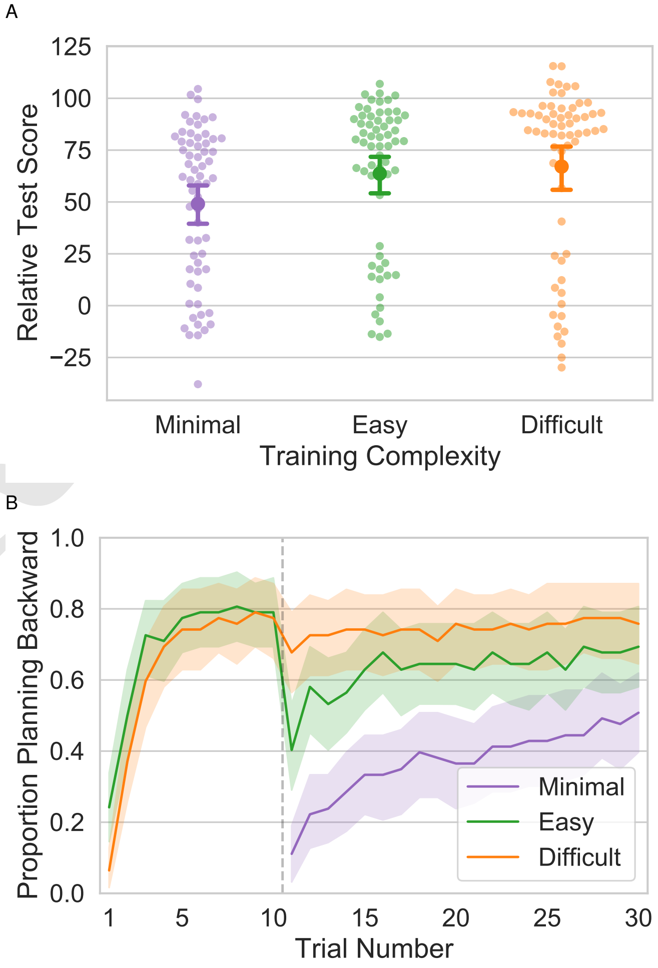

Fig. S6. Results of Experiment 8. (A) Average score in the test block for each of three levels of training complexity (see Figure S11). Each dot is the average score of one participant. (B) Proportion of participants who used a backward planning strategy as a function of the trial number. The first 10 trials were performed in the training environment. The last 20 trials were performed in the transfer environment (test block). For the Simple training environment, there is no distinction between backward and forward planning, so we omit these trials from the plot.

Analysis of verbal responses. To probe people's mental representation of the strategies they had learned, Experiments 1 and 6 asked participants "What have you learned? What are you doing differently now from what you were doing at the beginning of this training session?". To analyse our participants' verbal responses to this question, we categorized each response as either expressing one of ten types of lessons, or as expressing the belief of having learned nothing, or as failing to answer the question. We also recorded whether the lesson was formulated in a task-specific manner or in general terms that are applicable to the real world. We excluded 30 participants from the analysis of Experiment 6 because their descriptions of what they had learned were not properly recorded due to a technical error.

The proportion of each type of lesson among participants who received optimal metacognitive feedback versus no feedback is shown in Figure S7. When aggregating data from both the experiments and comparing the effects of optimal metacognitive feedback versus no feedback, we found significant difference in the proportions of participants who learned a general lesson (4.17% vs. 0.0%, $Z = 2.92$, $p = .004$) or a general beneficial lesson (3.28% vs. 0%, $Z = 2.58$, $p = .010$) but no significant difference in either the proportion of participants who learned a beneficial lesson (76.4% vs. 73.2%, $Z = 0.82$, $p = .410$), the optimal lesson (47.2% vs. 42.4%, $Z = 1.06$, $p = .288$), or a general version of the optimal lesson (0.30% vs. 0.0%, $Z = 0.77$, $p = .442$). These results might suggest that many participants had only a very limited awareness of the essence of the strategies they had learned. This would be consistent with the interpretation that they primarily relied on implicit learning.

For Experiment 6, we found that out of those participants who were trained with reinforcement a larger proportion reported having learned the optimal strategy compared to participants who trained without feedback (55% vs. 43%, $Z = 2.28$, $p = .023$) whereas being trained with information about the optimal strategy had no such effect (49% vs. 50%, $Z = -0.15$, $p = .880$). This is consistent with the finding that reinforcement is more effective at promoting strategy discovery than information as reported in the Main Text. When all beneficial lessons were considered simultaneously, neither reinforcement (81% vs. 74%, $Z = 1.74, p = .081$) nor information (79% vs. 76%, $Z = 0.88$, $p = .379$) had a statistically significant effect on the proportion of participants who reported one or more of them.

SI Methods

Metalevel MDP and dynamic programming. A metalevel MDP formalizes computation (e.g. planning) as a sequential decision process with computational actions that affect belief states. The belief state encodes a distribution over parameters of the environment, which determine the value of acting according to some policy. The reasoner sequentially selects computations that update this belief state, until ultimately deciding to take action by selecting the policy that has highest expected value given the current belief state.

The metalevel MDP for Mouselab-MDP can be formally represented by the tuple $(\mathcal{B}, \mathcal{C}, T_{\text{meta}}, r_{\text{meta}})$ where each belief $b \in \mathcal{B}$ encodes k Categorical distributions over the rewards that can be attained at each of the k non-initial states (in the training task shown in Figure 1, $k = 12$). In the initial belief state, these distributions are those that the rewards are drawn from (participants were informed of and quizzed on this knowledge). The computations, $\mathcal{C}$, include each of the k possible clicks as well as the termination operation, $\perp$, which executes the sequence of moves that has the highest expected value given the current belief state.

The metalevel transition function describes how computations update beliefs. Informally, executing computation i sets b_i to a discrete delta distribution that puts all probability mass on a single value that is sampled from b_i. All b_i are initialized to discrete uniform distributions that assign a probability of $1/4$ to the four possible outcomes. The terminal action $\perp$ always transitions to a unique terminal state, $b_\perp$. Formally,

$$T_{\text{meta}}(b, c, b') = \begin{cases} 1 & \text{if } c = \perp \wedge b' = b_\perp \\ b_c(x) & \text{if } b'_c(x) = 1 \wedge \forall j \neq c: \ b'_j = b_j \\ 0 & \text{otherwise} \end{cases} \quad [1]$$

Finally, the metalevel reward function describes the cost and benefits of computation. The cost of computation (i.e. clicking) is captured by setting $r_{\text{meta}}(b, c) = -\lambda \, \forall c \in \mathcal{C} \setminus \{\perp\}$. For simplicity, we assume that λ is equal to the fee that participants pay for each click (i.e., \$1). This is a simplification because processing the acquired information incurs additional cognitive costs that may differ across people. The benefit of computation are given by the expected quality of the decision that is ultimately made,

$$r_{\text{meta}}(b, \perp) = \max_{\mathbf{t} \in \mathcal{T}} \sum_{i \in \mathbf{t}} \mathbb{E}[b_i], \quad [2]$$

where $\mathcal{T}$ is the set of possible trajectories $\mathbf{t}$ through the environment.

Having formalized planning as a metalevel MDP, we can use standard dynamic programming techniques to solve for the meta-level Q-function (Q_{meta}). Because there are a finite number of clicks you can make in Mouselab-MDP, we have a finite-horizon MDP that can be solved exactly using backwards induction [3].

Feedback messages. Figure S8 illustrates the feedback messages the cognitive tutor would show when people planned too little (a), planned too much (b), selected a sub-optimal planning operation (c), or selected an optimal planning operation or move (d).

For the cognitive tutors used in Experiments 1-3, the scaling factor used to calculate the delay penalty was $a = 1$. For the cognitive tutor for the constant-variance environment used in Experiment 4, we set $a = 40/9$ to match the delay penalty for acting without planning to the 42 second delay of the cognitive tutor used in Experiments 1-3. In all other experiments the value of a was the same as in Experiments 1-3.

Experiment 1. Participants received a base pay of \$0.50 plus a performance-dependent bonus of 1 cent for every \$5 they earned in the posttest block (average bonus: \$1.41). They began with an endowment of \$100, to minimize the chance that their score would ever fall below zero.

The instructions informed participants about how to move, how to collect information, the cost of information, the minimum time of 7 seconds per trial, the structure of the experiment, and the bonus. No participants were excluded from the analysis.

DRAFT

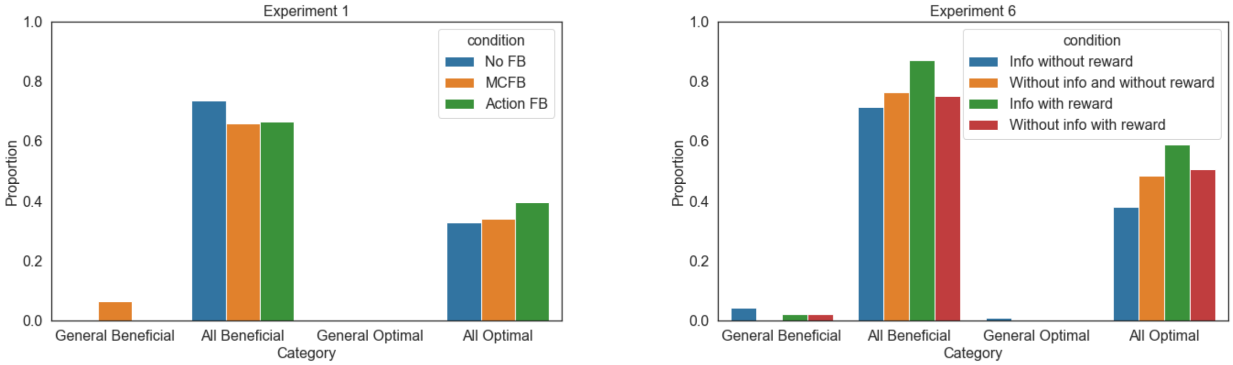

Fig. S7. Proportion of participants reporting having learnt different types of lessons by experiment and experimental condition. *General Beneficial*: participants who reported one of the beneficial lessons in a formulation that is general enough to be applicable in the real world. *All Beneficial*: participants who reported a beneficial lesson in a task-specific or in a generalized wording. *General Optimal*: participants who reported the optimal lesson in a formulation that is general enough to be applicable in the real world. *All optimal*: participants who reported the optimal lesson in a task-specific or general wording.

Experiment 2. Participants received a base pay of $2.50 and a performance-dependent bonus (average bonus: $0.47).

As in Experiment 1, the instructions explained how to move, how to collect information, the cost of information, and the minimum time of 7 seconds per trial.

The exit survey was the same as in Experiment 1 and we analyzed them in the same way. No participants were excluded from the analysis.

Experiment 3. Participants were paid a base pay of $3.00 plus a performance-dependent bonus (average bonus: $0.48).

The first HIT asked workers to only start the first part if they were certain they would also participate in the second part. The monetary incentives were set up to discourage completing only in the first part. The base pay of the first HIT was only $1. Participants were motivated to return to second part by an additional $2 baseline bonus that they would receive if and only if they completed both parts and the chance to earn a performance-dependent bonus proportional to their score in Part 2. Furthermore, participants could sign up for an email reminder that was sent the following day after the HIT for Part 2 had been posted. Participants could start the second HIT no sooner than 24 hours after they had started Part 1.

At the end of Stage 2, an exit survey asked participants what they had learned, their age, and their gender identity. All participants who completed both parts were included in the analysis.

Experiment 4. Participants received a base pay of $2.50 and a performance-dependent bonus (average bonus: $0.50). No participants were excluded from the analysis.

Experiment 5. Participants received a base pay of $3. The average bonus was $0.90. Demographic information about age was available from 526/1380 participants. Demographic information about gender was available from 525/1380 participants. Out of the 1380 participants who started the experiment only 42 dropped out during the experiment (13.2%). The drop out rate was very similar across all four conditions: 13.7% in the control condition, 13.9%, 13.4%, 11.7% in the experimental conditions without feedback, with action feedback, and with metacognitive feedback, respectively. No participants were excluded from the analysis.

The general instructions informed participants that they would perform a series of two different tasks. All participants were told that they would be able to use the strategy they learn in the training block to earn a bonus for their performance in the second task.

The video shown to the control condition can be found at www.youtube.com/embed/N7SDVVbxgNU. To ensure that participants paid attention, the experiment asked them to confirm that they were still watching the video at five random points during the video. If a participant failed to respond to one of these questions within 20 seconds the video would start again from the beginning.

The transfer prompts asked all participants to articulate which planning strategy they found to work best in the Web of Cash task ("What can you do to increase your chances of finding a good path without spending too much on clicks?" and "How would you describe the lesson that you learned to a friend who wants to use the art of *Web of Cash* to their own life?"), reflect on for which kinds of problems this strategy is effective ("What is an example of a decision from your own life where this strategy might work especially well?" and "In which ways is this situation similar to the *Web of Cash* game?"), and how this strategy might be used to plan a road trip ("In the second part of this HIT you will play the Travel Agent game. In this game you play a travel agent who wants to find an inexpensive route to one of the airports on a large island. The route will pass through multiple cities, and the client must spend the night in a hotel in each one, including the final city that houses the airport. The travel agent can find out the price of the least expensive hotel in each city, but this is a time consuming process and they would like to plan the trip as quickly as possible. How might you use what you have learned from Web of Cash to do well in the Travel Agent game?").

The transfer block is available at https://roadtriptwo.herokuapp.com. It comprised instructions, a quiz about the instructions, and eight rounds of the Road Trip task. The instructions asked the participant to imagine themselves as a travel agent who has to plan a client's road trip from a very expensive interview cafe that charges them $1 per second.

DRAFT

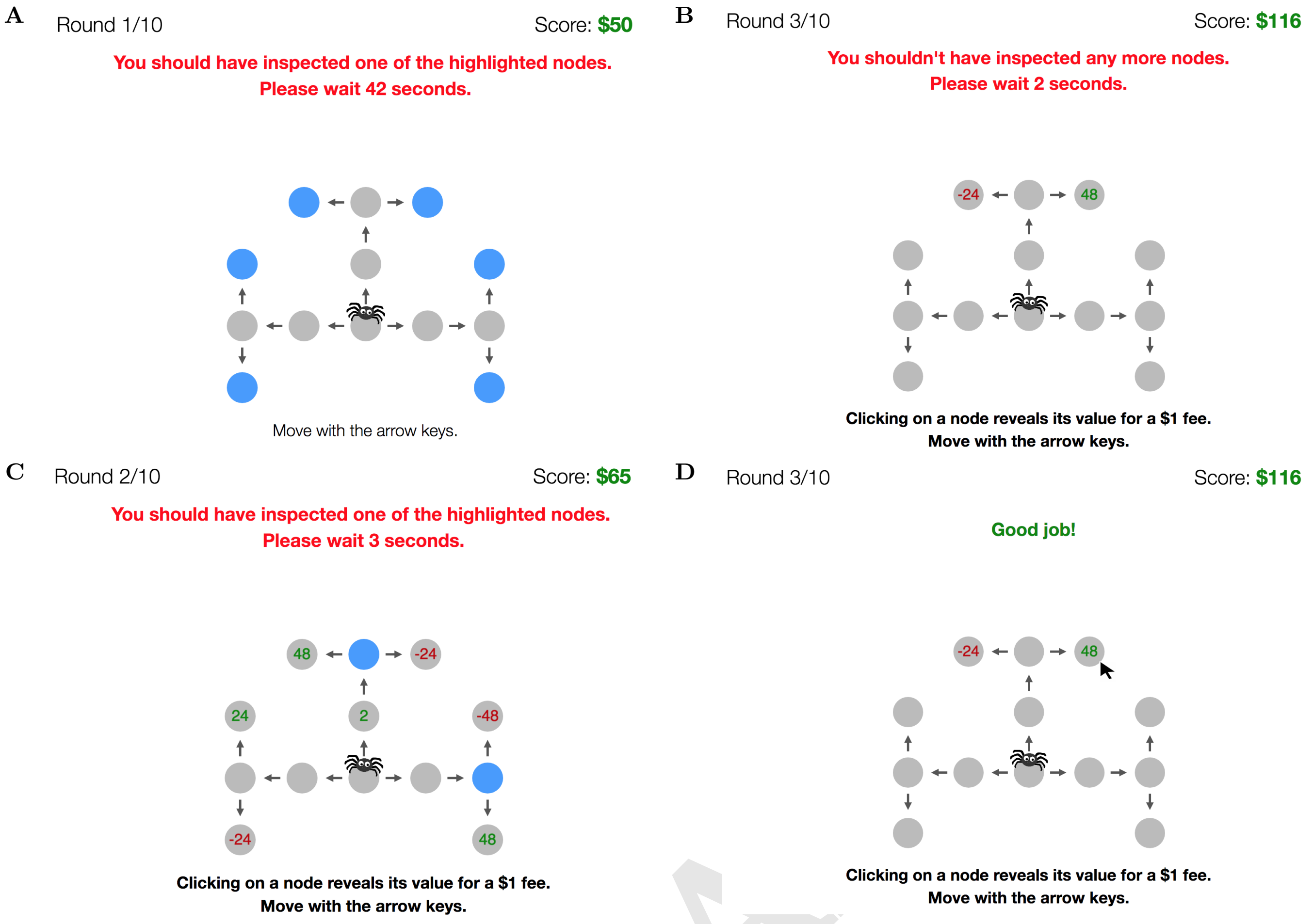

Fig. S8. Examples of feedback messages that the cognitive tutor displays when the user planned to little (a), planned too much (b), selected a sub-optimal planning operation (c), or selected an optimal planning operation or move (d).

The instructions further informed participants about the rules, mechanics, and financial incentives of the game. In addition, the participants were explicitly told that they do not have to research the prizes of all hotels along the trip. Participants who answered one or more quiz questions incorrectly were required to reread the instructions and retake the quiz until they answered all questions correctly.

Each trial of the Road Trip task presented participants with a new road map with a different layout, different cities, and different hotel prizes. Each map included between 2 and 4 airport cities that could be reached in 3 to 5 steps. On each trial participants started out with a budget of $350. They were charged $1 for each second that they invested in planning. To plan the trip participants could type the names of the cities on the map into a search bar. After “searching” for four seconds, the price of the hotel at the searched location was revealed on the map. Participants specified the road trip by clicking on individual roads in any order they like and could undo and update their selection until they chose to submit their solution. The sum of hotel prices along the selected route was subtracted from their budget. Participants received a performance dependent bonus of 1 cent for every $10 that were left on their budget.

The hotel prices of airport cities were uniformly distributed over the possible values of $40/night, $80/night, $120/night, and $160 whereas the hotel prices in other cities were either $30/night, $35/night, $40/night, and $45/night with equal probability.

Experiment 6. Participants received a base pay of $0.50. The average duration of the experiment was 16.15 ± 6.54 min. The average bonus was $1.05. In order to independently manipulate the presence of information and reward, we changed both the visual display and timing of the feedback, as described below.

Visual demonstrations of the optimal strategy. For conditions with information, the feedback instructed participants what the optimal strategy would have done when the participant made an error. When the optimal strategy would have made a click that the participant did not make, a clicking icon appeared at the top of the screen and the location(s) that the optimal strategy might have clicked on were highlighted in blue. When the optimal strategy would have started moving but the participant continued clicking, an arrow keys icon appeared. The instructions informed the participant that the click icon meant that “An expert player would have inspected one of the highlighted nodes.” and that the arrow keys icon meant that “An expert player would have stopped clicking and started moving.”. The quiz ensured that participants understood this before they were allowed to start the experiment. To control

for the difficulty of the quiz, the conditions without information instead received two alternative questions ("When can you use the node inspector?" and "What happens when you click on a node?"). The different types of feedback messages are shown in Figure S10.

Timing. To ensure that participants in the conditions with information had an opportunity to process the feedback, we imposed a one-second delay after every click. During this period, a question mark appeared on the inspected location to visually confirm the participant's action, and feedback was shown at the top of the screen. To minimize differences between conditions, the delay and question mark were retained in the conditions without information. In conditions with reward, the delay penalty was appended to the standard one second delay and the value of the inspected node was not revealed until the end of the combined delay. To control for the amount of time that participants had to reflect on the outcomes of their planning, participants in the non-reward conditions had to wait 11 seconds (one plus the mean delay per trial in Experiment 1) after each trial while the screen said "Preparing next trial..." whereas participants in the reward condition had to wait for only one second.

No participants were excluded from the analysis.

Experiment 7. Experiment 7 examined to which extent people's propensity to transfer the strategy taught by the cognitive tutor to a new environment depends on how well the strategy is adapted to the structure of the new environment. All participants began with a training block identical to the metacognitive feedback condition of Experiment 1. The test block varied by condition. The control group was tested in the same task environment but without feedback (as in the test block for Experiment 1). For the experimental group, the environment changed without indication to the uniform environment used in Experiment 4

We recruited 102 participants on Amazon Mechanical Turk (average age 34.78 years, range: 18–71 years; 43 female). 51 participants were assigned to the experimental condition and 51 participants were assigned to the control condition.

Participants received a base pay of \$0.50 and a performance-dependent bonus (average bonus: \$1.43). No participants were excluded from the analysis.

Experiment 8. Experiment 7 investigated how the transferable benefits of training with the cognitive tutor depend on the complexity of the training environment. The experiment comprised a training block with 10 trials and a test block with 20 trials. In the training block, participants practiced planning with a cognitive tutor that, depending on the experimental condition, taught them the optimal strategy for a minimal, easy, or difficult task with 1, 2, and 3 steps, respectively (see Figure S11). In the test block, all participants were tested on the 5-step transfer task introduced in Experiment 2. In the *minimal* training task, the three nodes rewards were independently drawn from a discrete uniform distribution over the possible values $\{-48, -24, +24, +48\}$. In the *easy* task, the rewards at the first level were drawn from a uniform distribution over the possible values $\{-4, -2, +2, +4\}$ and the rewards at the second level were drawn from a uniform distribution over the possible values $\{-48, -24, +24, +48\}$. In the *difficult* training environment participants had to choose a series of three actions; this task was identical to the training environment used in Experiments 1-3.

We recruited 187 participants on Amazon Mechanical Turk (average age 33.67 years, range: 18–67 years; 70 female). 63 participants were assigned to the minimal condition, 62 to the easy condition, and 62 to the difficult condition.

Participants received a base pay of \$0.50 and a performance-dependent bonus (average bonus: \$1.28). No participants were excluded from the analysis.

Analysis of verbal responses. To probe people's mental representation of the strategies they learned, each experiment asked them "What have you learned? What are you doing differently now from what you were doing at the beginning of this training session?". To analyse our participants' verbal responses to this question, we developed a rubric categorizing the responses according to the lesson learned and whether it was specific to our task (e.g., click on the third layer) or general enough to be applicable in everyday life (e.g., prioritize long-term consequences). We trained three raters to apply this rubric until all three of them completely agreed on the categories for 16 out of 20 examples they had rated independently (80%). This training process comprised several iterations in which the raters discussed their disagreements and then codified their resolutions in a revised version of the rubric. The data from the four experiments was then distributed across the four raters so that each response had to be categorized by only one of them. The final version of the rubric assigned each response to one of the following categories:

1. Nothing: The participant reports having learnt nothing (e.g., "Nothing.").
2. The participant did not answer the question (e.g., "asdf").
3. The participant reports having learnt to set goals (e.g., "Search for a \$48 reward"), to find out about long-term consequences (e.g., "Click on the nodes at the end."), or to prioritize long-term outcomes over immediate and short-term rewards (e.g., "It doesn't matter if I lose \$2 in the first step or not. What matters is what I get in the third step.").
4. The participant reports having learnt to satisfice (e.g., "I learned to take the first path I can find that pays at least \$24.").
5. The participant reports having learnt to consider multiple options (e.g., "I learned to click on nodes from multiple different paths.").
6. The participant reports having learnt to take more risks (e.g., "I learned to take a path without knowing all of the rewards/losses along the way.").
7. The participant reports having learnt something about the game itself (e.g "This game is hard".) or some property of the game (e.g "There are as many negative values as there are positive values.").
8. The participant reports having learnt to invest less into planning (e.g., "to minimize my clicks").

DRAFT

9. The participant reports having learned how to plan without reporting a specific strategy ("I learned to develop a strategy.").

10. The participant reports having learned something about themselves (e.g., "I should come up with a better strategy next time.").

11. The participant reports having learned how to tradeoff the costs of planning against its benefits (e.g., "I would reveal a node to get a better reward because it costs only $1.").

12. The participant reports having learned something else.

For each lesson participants reported to have learned we additionally recorded whether it was specific to our task (e.g., "Click on the nodes at the end.") or general enough that it could be applied in the real world (e.g., "Think about the long-term consequences."). In subsequent analyses we grouped lessons 1, 3, 5 , 7, 8, and 9 together into a category for beneficial lessons.

References

1. Jain YR, Callaway F, Lieder F (2019) Measuring how people learn how to plan in *CogSci 2019*, eds. Goel A, Seifert C, Freksa C. (Cognitive Science Society, Austin, TX).
2. Jain YR et al. (2021) A computational process-tracing method for measuring people's planning strategies and how they change over time. *Preprint*.
3. Puterman ML (2014) *Markov Decision Processes.: Discrete Stochastic Dynamic Programming*. (John Wiley & Sons).

DRAFT

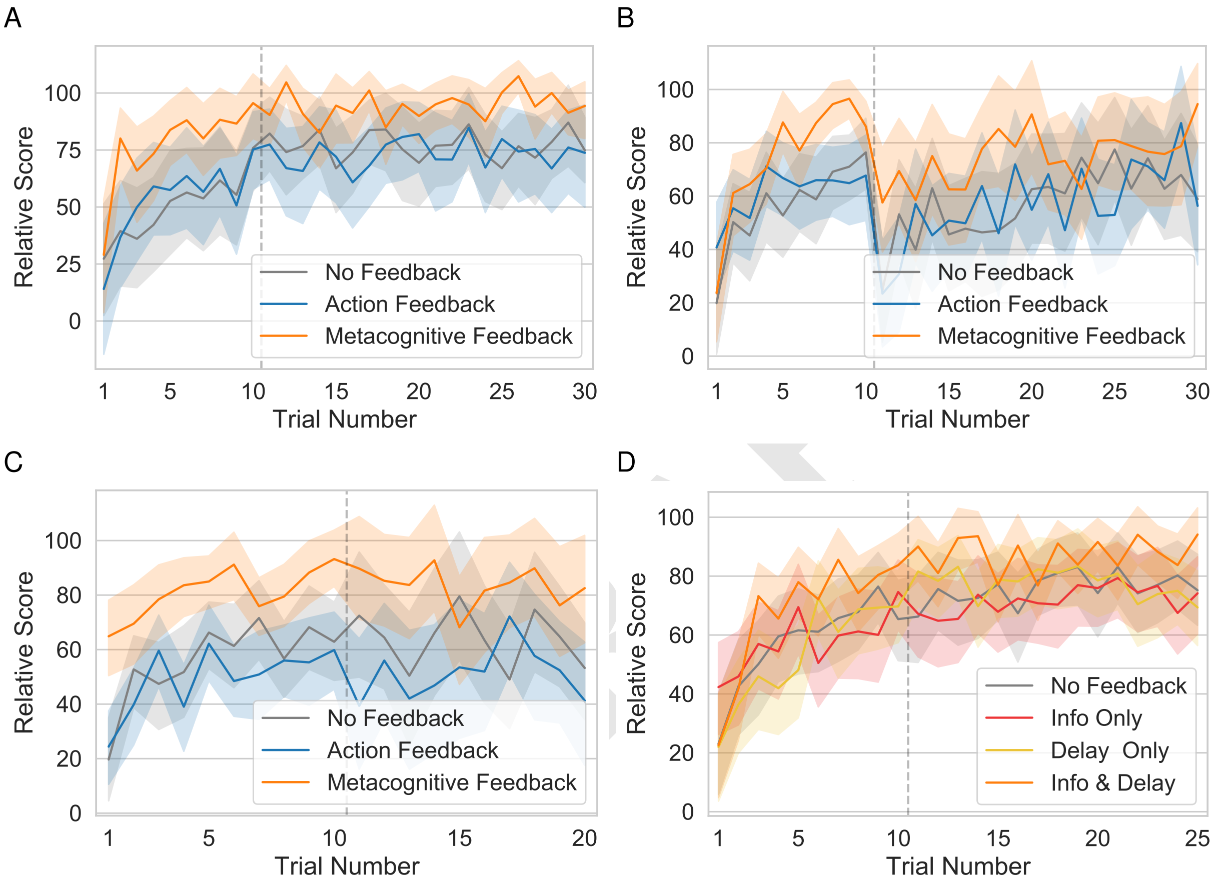

Fig. S9. Learning curves. Average performance in each experiment (A=1, B=2, C=3, D=6) by experimental condition and trial number. The vertical line marks the end of the training block and the beginning of the test block.

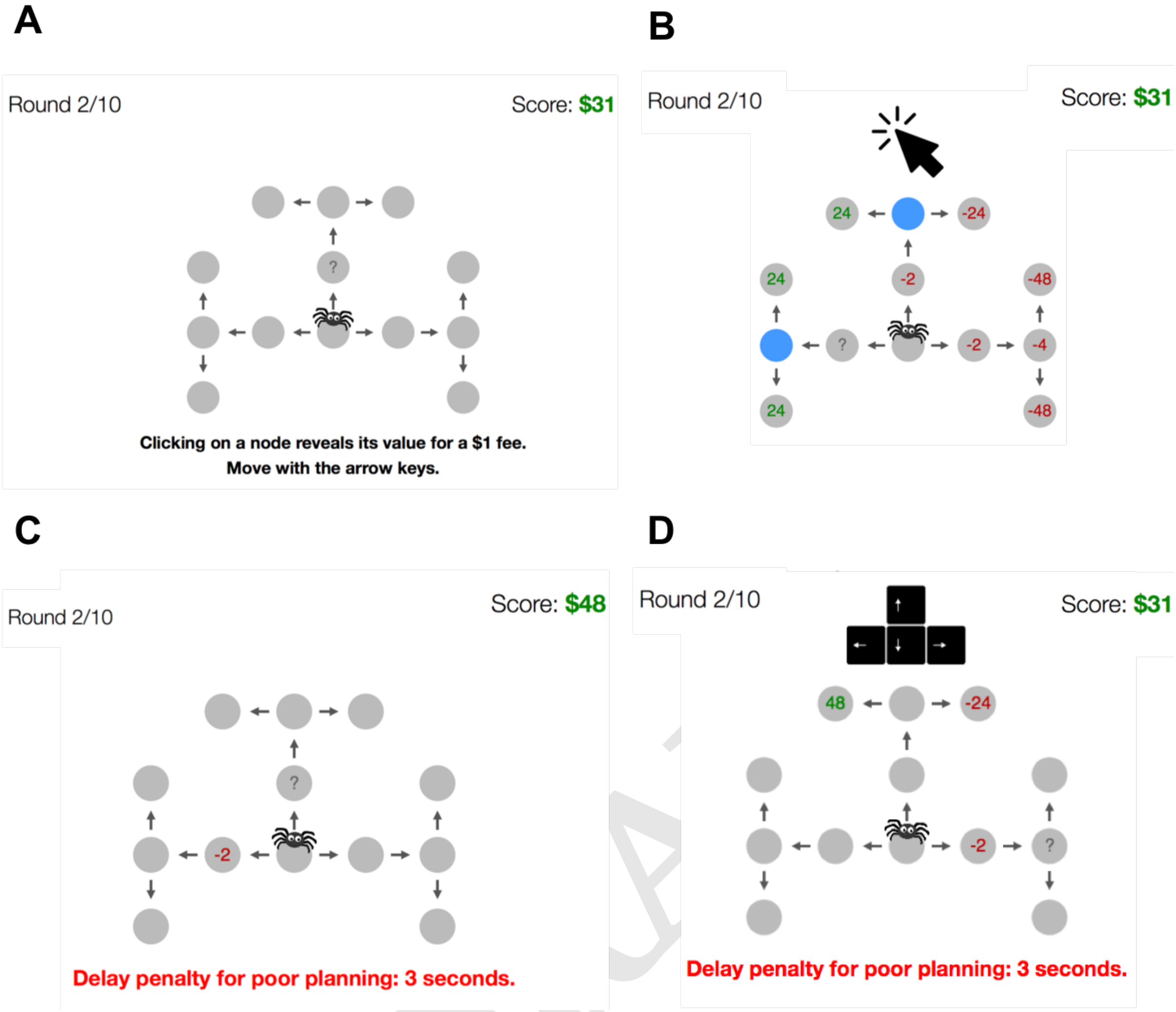

Fig. S10. Feedback in the four conditions of Experiment 6. A: No information and no reinforcement. B: Information and no reinforcement. C: No information and reinforcement. D: Information and reinforcement.

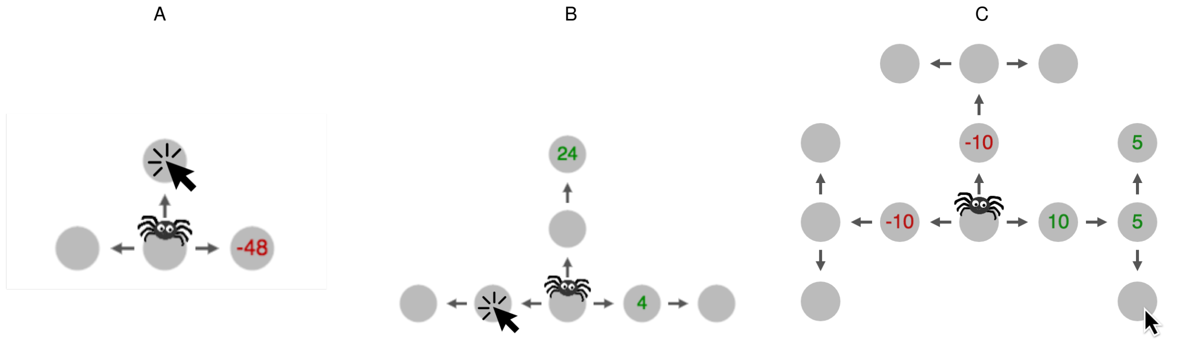

Fig. S11. Training environments used in the three experimental conditions of Experiment 8. A: minimal training environment (1 step). B: simple training environment (2 steps). C: difficult training environment (3 steps).
